# Subtle Alterations in PCNA-Partner Interactions Severely Impair DNA Replication and Repair

**DOI:** 10.1371/journal.pbio.1000507

**Published:** 2010-10-12

**Authors:** Yearit Fridman, Niv Palgi, Daniel Dovrat, Shay Ben-Aroya, Philip Hieter, Amir Aharoni

**Affiliations:** 1 Departments of Life Sciences and the National Institute for Biotechnology in the Negev (NIBN), Ben-Gurion University of the Negev, Be'er Sheva, Israel; 2 The Nano Center, The Mina and Everard Goodman Faculty of Life Sciences Bar-Ilan University, Ramat-Gan, Israel; 3 Michael Smith Laboratories, University of British Columbia, Vancouver, British Columbia, Canada; Brandeis University, United States of America

## Abstract

The robustness of complex biological processes in the face of environmental and genetic perturbations is a key biological trait. However, while robustness has been extensively studied, little is known regarding the fragility of biological processes. Here, we have examined the susceptibility of DNA replication and repair processes mediated by the proliferating cell nuclear antigen (PCNA). Using protein directed evolution, biochemical, and genetic approaches, we have generated and characterized PCNA mutants with increased affinity for several key partners of the PCNA-partner network. We found that increases in PCNA-partner interaction affinities led to severe in vivo phenotypic defects. Surprisingly, such defects are much more severe than those induced by complete abolishment of the respective interactions. Thus, the subtle and tunable nature of these affinity perturbations produced different phenotypic effects than realized with traditional “on-off” analysis using gene knockouts. Our findings indicate that biological systems can be robust to one set of perturbations yet fragile to others.

## Abstract 2

Dynamic switching of PCNA-partner interactions is essential for normal DNA replication and repair in yeast.

## Author Summary

Many biological processes are mediated by complex protein-protein interaction networks. The most highly connected proteins in such networks, termed hub proteins, precisely regulate biological processes by the regulated and sequential binding and releasing of partner proteins. In the case of DNA replication and repair, proliferating cell nuclear antigen (PCNA) is a hub protein that encircles the DNA to dynamically bind and release a variety of DNA-modifying enzymes. In this work, we explored the impact of subtle alterations of PCNA-partner interaction affinities on DNA replication and repair in yeast. Using directed evolution approaches, we generated a large library of PCNA mutants and selected for those with enhanced affinity for five different PCNA partners. In vivo analysis of such mutants indicated the high sensitivity of DNA replication and repair processes to minor alterations in PCNA-partner interaction affinities. Importantly, we discovered that some of the defects observed in the strains with increased PCNA-partner protein interaction far exceed the defects observed when the same partner protein is deleted altogether. Our analysis suggests that the cost of misregulating biological processes through disruption of the carefully orchestrated action of hub-interacting proteins can be much higher than the cost of deleting parts of the network altogether, demonstrating both the fragility and robustness of biological processes.

## Introduction

Robustness, the ability to maintain performance in the face of environmental and genetic perturbations, is a fundamental trait of biological processes [1]–[5]. Accordingly, many design principles ensuring the robustness of biological processes, such as redundancy, modularity, and feedback mechanisms, have been described [2],. However, robustness to one class of perturbations can render the same system fragile to other classes of perturbation. The concept of robust yet fragile is a well-known feature in the field of engineering and is one of the most common properties of complex systems [1],[2]. In the case of complex biological processes, by contrast, very little is known regarding perturbations that result in enhanced sensitivity or fragility of a process. Understanding such perturbations could provide new mechanistic insight into biological processes mediated by complex hub-partner interactions and could elucidate relationships between the robustness and fragility of biological processes.

In eukaryotes, DNA replication and repair processes are mediated by the proliferating cell nuclear antigen (PCNA) through the recruitment of various DNA-modifying enzymes to the replication fork [8], including members of different families of DNA polymerases, helicases, exonucleases, and ligases [9]–[11]. PCNA forms a sliding platform to enhance the processivity and catalytic activity of many DNA-modifying enzymes by tethering them to the DNA template. Remarkably, many of the PCNA partners interact with a particular loop on PCNA through a conserved binding motif, suggesting that these partners bind and dissociate sequentially in order to perform their particular function. Switching of partners on the PCNA platform is crucial during different stages of DNA replication and repair, such as lagging strand replication, translesion synthesis (TLS), mismatch repair (MMR), and base excision repair (BER) [8]. In recent years, post-translational PCNA modifications have been shown to be an important control mechanism regulating partner switching on PCNA during DNA repair processes [12],[13].

To investigate the importance of PCNA-partner interactions for DNA replication and repair, previous studies have focused on abolishing these interactions via mutational approaches [14]–. However, due to the functional redundancy exhibited by PCNA partners [17], abolishing such interactions often results in relatively minor phenotypic defects. Hence, an alternative approach to study the regulation of PCNA-partner interactions during DNA replication and repair involving systematically strengthening specific PCNA-partner interactions is required. Due to the competitive nature of binding to PCNA, strengthening PCNA-partner interactions could result in prolonged PCNA-partner association, thereby hindering the binding of other partners required for the replication and repair processes. Tighter PCNA-partner interactions cannot, therefore, be suppressed by functional redundancy within the pool of network proteins and can thus reveal the importance of accurate regulation of PCNA-partner interactions for the progression of DNA replication. On the system level, this approach could shed new light on the robustness or fragility of DNA replication and repair in the face of such perturbations. In recent years, protein engineering methodologies, including directed protein evolution [18],[19], have proven to be highly effective for the generation of proteins with increased binding affinity for target protein partners [20].

**Figure 1:**
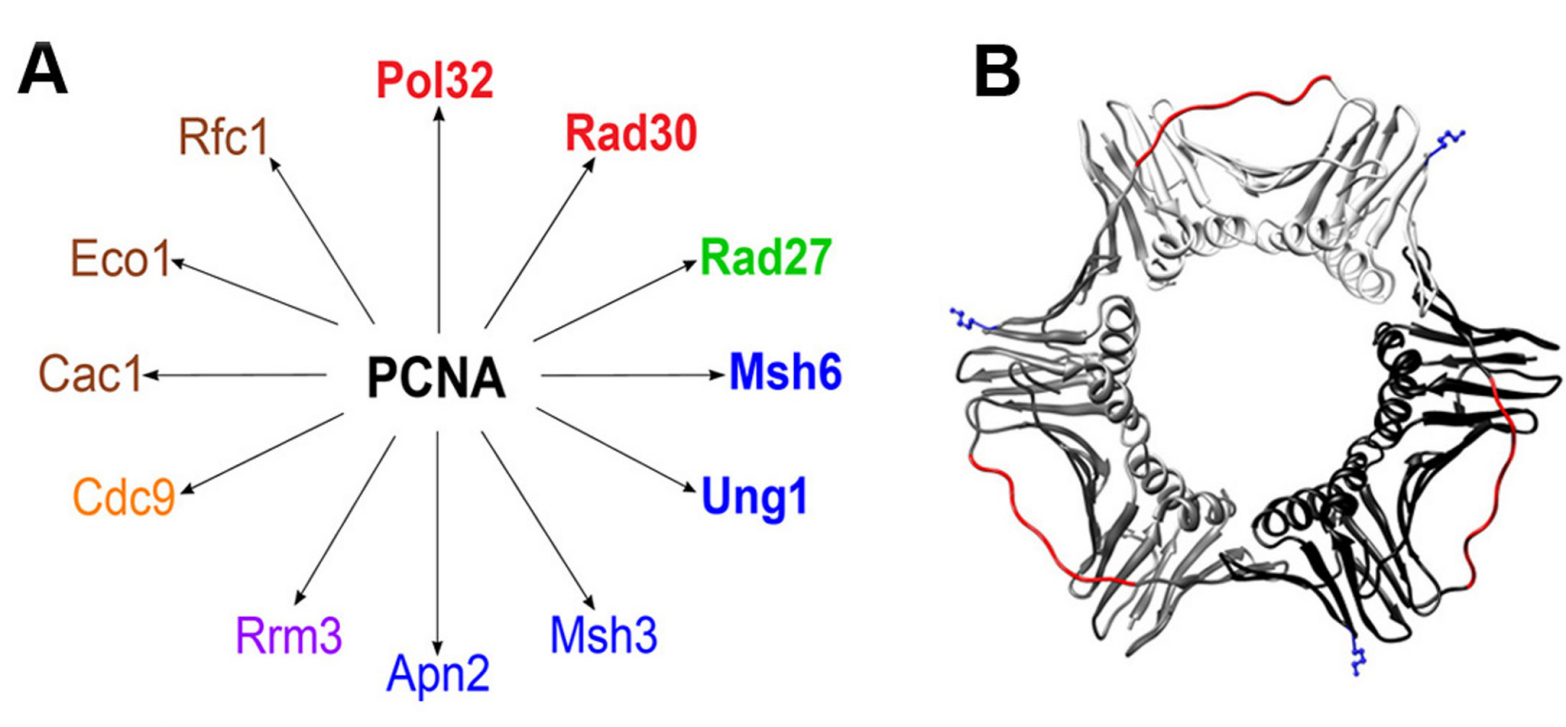
PCNA-partner interaction network mediating DNA replication and repair and PCNA structure. (A) PCNA-partner interaction network containing the 12 PCNA partners containing PCNA-interacting protein (PIP) used in this study, including DNA polymerases (Pol32 and Rad30, red), an endonuclease (Rad27, green), partners involved in DNA repair (Msh3, Msh6, Apn2 and Ung1, blue), a helicase (Rrm3, purple), a ligase (Cdc9, orange), and partners involved in various processes, including loading PCNA onto DNA (Rfc1, brown), sister chromatid cohesion (Eco1, brown), and chromatin acetylation (Cac1, brown). PCNA was evolved for higher binding affinity for Pol32, Rad30, Rad27, Msh6, and Ung1 (highlighted in bold). (B) Model of the PCNA structure (PDB code:1PLQ) [25]. Each monomer is shown in white, grey, or black. The IDCL loop is highlighted in red and the K164 residue is highlighted in blue. The model was generated using the UCSF chimera program.

**Figure 2:**
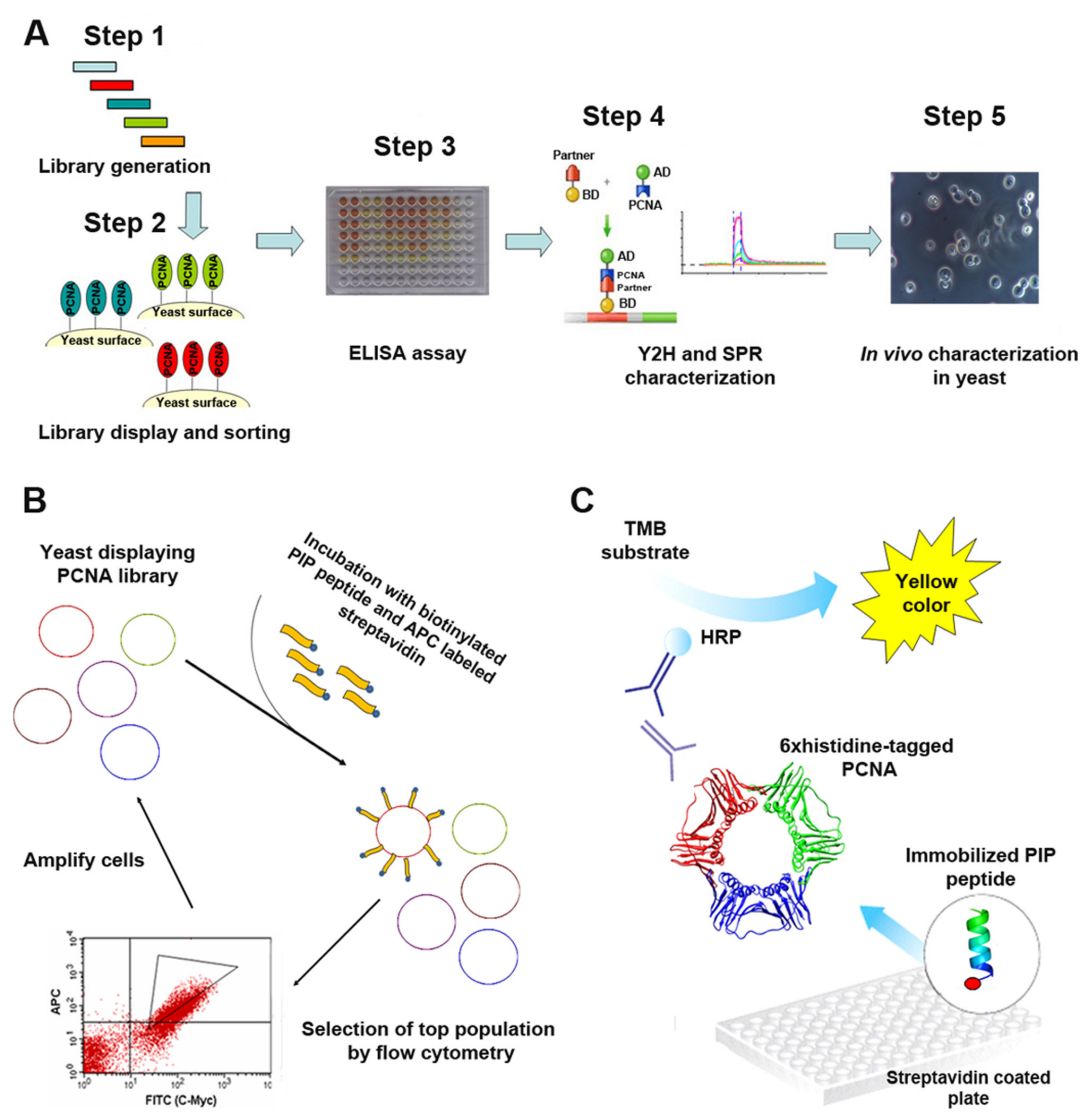
Work-flow for the generation and characterization of PCNA mutants with enhanced affinity for different partners. (A) Overview of the workflow, including generation and sorting of PCNA libraries displayed on the yeast cell surface (Step 1 and 2) and subsequently subjecting the enriched libraries to ELISA to identify improved mutants (Step 3). Binding characterization of the selected PCNA mutants by the yeast two hybrid system (Y2H) and surface plasmon resonance (SPR) is next performed (Step 4). Finally, in vivo characterization of the PCNA mutants in yeast cells is conducted (Step 5). (B) Detailed description of the selection process of the PCNA library displayed on the yeast cell surface for PCNA mutants with improved affinity for PIP peptides derived from the different partners, relying on yeast surface display (YSD) and flow cytometry (Step 2). (C) Detailed description of the ELISA assay used for the detection of PCNA-PIP peptide interactions (Step 3). Target biotinylated PIP peptides derived from the different partners (see Figure S1 for sequences) are immobilized on streptavidin-coated plates. *E. coli* cell lysates containing 6×histidine-tagged mutant PCNA are applied to the plates. Following washing, primary and secondary antibodies are added to facilitate the detection of PCNA binding to the PIP peptides.

In this study, we have examined the robustness or fragility of PCNA-mediated DNA replication and repair processes in the face of perturbations altering PCNA-partner interaction affinities. To do so we have generated and thoroughly characterized a collection of novel PCNA mutants exhibiting higher binding affinities for five different partners taking part in a variety of PCNA-mediated processes, including Pol32, Rad27, Rad30, Msh6, and Ung1, participating in lagging strand replication, TLS, MMR, and BER, respectively (Figure 1). Surprisingly, in vivo analysis of these mutants revealed strong replication defects, indicating the high sensitivity of these processes to increases in PCNA-partner interaction affinities. Moreover, these defects illustrate the importance of a fine balance between different PCNA-partner interaction affinities for the progression of DNA replication and repair. The generation and in vitro and in vivo characterization of the PCNA mutants were performed using a newly developed integrated platform that includes directed evolution, biochemical, and genetic approaches (Figure 2).

## Results

### The Experimental Approach

To generate PCNA mutants with enhanced affinity for different partners exhibiting a variety of DNA-modifying activities (Figure 1), we utilized directed evolution methodologies. Directed evolution experiments are based on the principles of natural Darwinian evolution and consist of two major steps: (i) creation of genetic diversity in the target gene in the form of gene libraries and (ii) effective selection or screening of those libraries for the desired activity [19],[21]. Accordingly, we first generated a large PCNA mutant library and displayed this library on the yeast cell surface (Figure 2A, Step 1) [22]. To enrich the PCNA library for mutants with enhanced affinity for the target partner, the displayed PCNA library was incubated with biotinylated peptide derived from the target partner (see below) and streptavidin-conjugated allophycocyanin (APC), in addition to a fluorescent antibody against the myc-tagged PCNA. The top fluorescent cell population was selected by fluorescence-activated cell sorting (FACS; Figure 2A, Step 2 and Figure 2B). Next, the enriched libraries were sub-cloned, expressed, and screened in *E. coli* cells for mutants showing enhanced affinity for the target partners, using an enzyme-linked immunosorbent assay (ELISA). The ELISA experiment for the detection of PCNA-PIP peptide interactions was performed with crude *E. coli* cell lysates containing the different mutants incubated with biotinylated PIP peptide-coated plates. The amount of bound PCNA was analyzed using antibodies against the 6×histidine-tagged PCNA (Figure 2A, Step 3 and Figure 2C). To further characterize the binding profile of selected PCNA mutants toward an array of partners, yeast two hybrid (Y2H) [23] and surface plasmon resonance (SPR) [24] assays were used (Figure 2A, Step 4). Finally, to examine the in vivo activities of the selected PCNA mutants, these were reintroduced as a sole source of PCNA into yeast cells using plasmid shuffle of centromeric plasmids in a strain lacking the chromosomal *POL30* gene. The resulting strains were subjected to a variety of DNA replication and repair assays (Figure 2A, Step 5).

### Generation and Enrichment of the PCNA Library Using Yeast Surface Display (YSD)

PCNA interacts with most of its partners through an inter-domain connecting loop (IDCL) that connects the two domains of the PCNA monomer (Figure 1B) [25]. Other sites of interactions include residues at the C-terminal and N-terminal regions of PCNA [26]–[29]. Accordingly, the majority of PCNA partners contain a conserved binding motif termed the PIP (PCNA-interacting protein) box, located in the N- or C-terminal region of the partner, distinct from its active site (see Figure S1 for peptide sequences) [9],[10]. To generate a large PCNA mutant library, we focused on the diversification of the IDCL region while maintaining the conserved IDCL residues constant due to their specific interactions (e.g. residues I126 and L128) with conserved residues in the PIP region [29],[30]. We hypothesized that the non-conserved residues may control the specificity of the different PCNA-partner interactions. Therefore, we fully diversified the I121, A123, F125, and E129 non-conserved positions (Figure S2), yielding a library including 160,000 different mutants.

To establish a high-throughput screening system for the detection of PCNA binding to PIP peptides derived from the different partners (Figure 1), we efficiently displayed PCNA capable of binding the target PIP peptides on the outer membrane of yeast cells using YSD methodology (Figure S3A) [22]. To confirm that the observed PIP peptide binding was a result of specific PCNA-PIP interactions, we tested the binding of WT PCNA to a mutated Rad30 PIP peptide [15], as well as the binding of an inactive PCNA mutant (i.e., PCNA79) [31] to the Rad30 PIP peptide (Figure S3B–D). In both experiments, a dramatic reduction in binding affinity was observed, indicating a specific interaction between the IDCL and the PIP peptide (Figure S3B–D).

**Figure 3:**
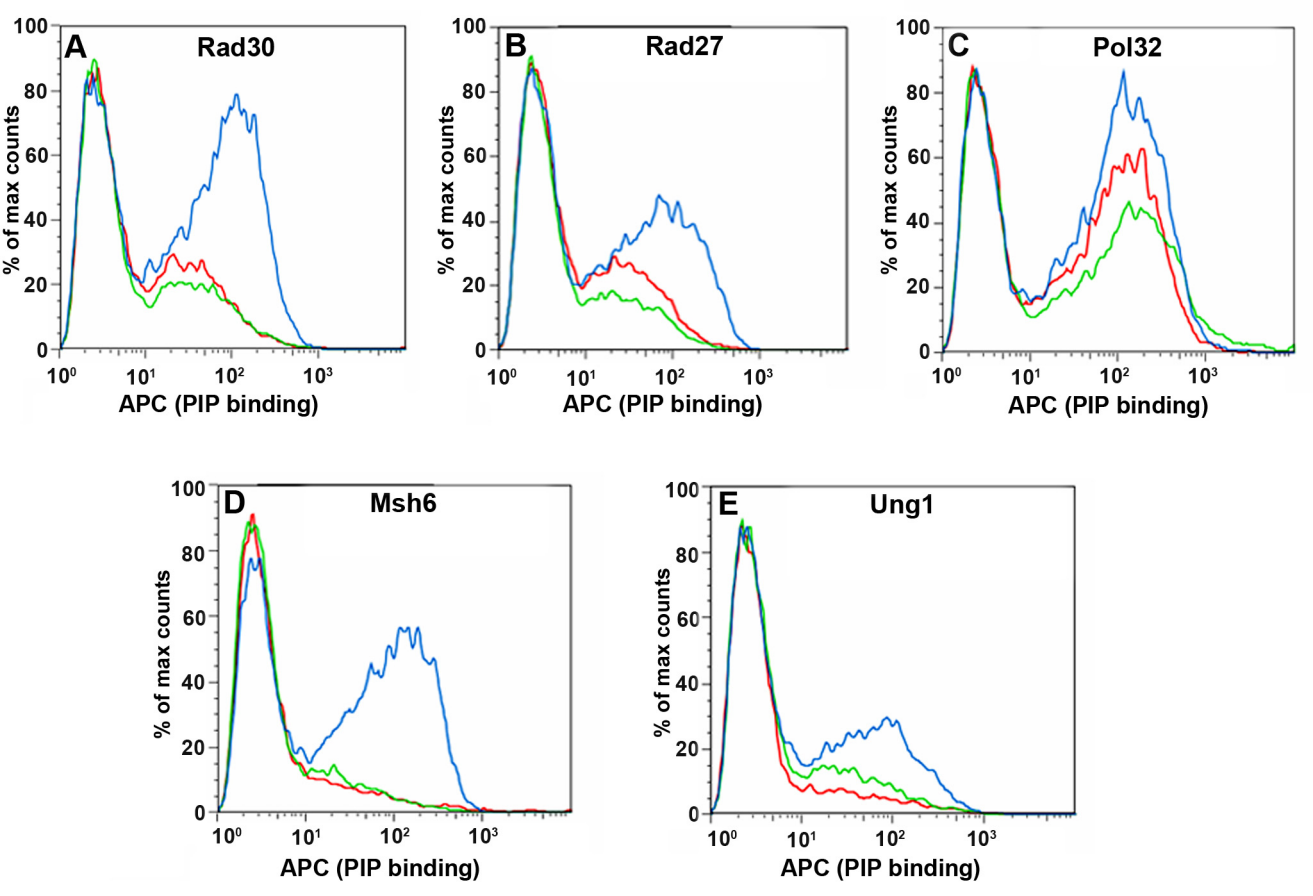
Enrichment of PCNA libraries displayed on the yeast cell surface for mutants exhibiting increased affinity for the target PCNA partners, as assessed by flow cytometry. FACS histogram analysis of cells displaying wild-type PCNA (red), a naïve library (green), and libraries following 3–5 rounds of enrichment (blue), following incubation with biotinylated PIP peptide derived from Rad30 (A), Rad27 (B), Pol32 (C), Msh6 (D), or Ung1 (E). Binding of PIP peptides was measured following subsequent incubation with allophycocyanin (APC)-labeled streptavidin (*x*-axis).

To enrich the PCNA library for PCNA mutants with enhanced affinity for the target partners (Figure 1A), yeast cells expressing the PCNA library were independently incubated with the five PIP peptides and more than 5×10^6^ cells were analyzed and sorted by FACS (Figure 2B). Three to five iterative rounds of enrichment were performed until a significant enrichment for PCNA mutants with increased affinity for all PIP peptides was obtained (Figure 3).

### Identification and Characterization of PCNA Mutants

To identify single PCNA mutants with enhanced binding affinity for target partners, the five FACS-enriched libraries (Figure 3) were sub-cloned into a bacterial plasmid, overexpressed in *E. coli*, and screened by ELISA (Figure 2C). Using this approach, the crude cell lysates of 10–20 clones from each of the five enriched libraries were screened for binding to a given PIP peptide. The top performing 2–4 mutants from each of the five ELISA screens (Table 1) were then taken for further in vitro and in vivo characterization, as described below.

**Table 1:** Summary of the in vitro and in vivo characterization of PCNA mutants.

PCNA (*POL30*) Mutant	IDCL Sequence^a^	Binding of PCNA Mutants to Target Partners^b^					Spontaneous Mutation Rate^e^ Can^R^ (Fold Increase)
		Rad30	Rad27	Pol32	Msh6	Ung1	
*POL30*	D**I**D**A**D**F**LKI**E**EL	1	1	1	1	1	1.5 [1.5, 2.2]×10^−7^
*pol30*-Rad30E2	D**D**D**W**DFLKI**L**EL	**1.4** c	**0.4** c	**0.4** c	1	1	1.5 [1.5, 2.0]×10^−6^ (10)
*pol30*-Rad30E9	D**N**D**W**DFLKI**D**EL	**1.6** c **(8.5)** d	**0.5** c	1	1	1	2.3 [1.7, 3.0]×10^−6^ (15.3)
*pol30*-Rad27E6	D**G**D**Y**D**I**LKI**R**EL	1	**1.5** c	1	1	1	1.2 [0.8, 1.4]×10^−6^ (8)
*pol30*-Rad27E31	D**G**D**Y**D**V**LKI**R**EL	1	**1.5** c	1	1	1	ND
*pol30*-Rad27L1^f^	D––**V**D**I**LKI**G**EL	**0.4**	**1.7** c	1	1	1	NA
*pol30-*Rad27L2^f^	D––**V**D**T**LKI**T**EL	**0.5**	**1.5** c	1	1	1	NA
*pol30*-Pol32E5	D**A**D**N**DFLKI**S**EL	1	1	**1.5** c	1	1	2.0 [1.4, 2.8]×10^−6^ (13.3)
*pol30*-Pol32E9	D**N**D**V**D**S**LKI**I**EL	1	**1.2**	**1.3** c **(2)** d	1	1	6.0 [4.7, 7.1]×10^−7^ (4)
*pol30*-Msh6E2	D**Y**D**R**D**M**LKI**S**EL	1	**1.3**	1	**1.4** c	1	1.1 [0.7, 1.6]×10^−6^ (7.3)
*pol30*-Msh6E6	D**Y**D**K**D**L**LKI**I**EL	1	**1.3**	1	**1.4** c **(1.6)** d	1	6.1 [5.1, 8.8]×10^−7^ (4.1)
*pol30*-Ung1E2	D**D**D**S**DFLKI**P**EL	1	1	1	1	**1.3** c	3.3 [2.2, 3.3]×10^−7^ (2.2)
*pol30*-Ung1E3	D**F**D**Y**D**E**LKIEEL	1	1	1	1	**1.3** c **(3.5)** d	3.1 [2.1, 3.9]×10^−7^ (2.1)
*pol30*-79	DIDADF**A**K**A**EEL	**0.4** c	**0.5** c	**0.3** c	**0.4** c	**0.3** c	3.3 [2.2, 5.1]×10^−7^ (2.2)

a The IDCL sequences of the PCNA mutants. The positions that were diversified in the naïve library and mutations identified in the selected mutants are marked in bold. The mutations in *pol30*-79 [31] are marked in bold.

b Binding characterization of the PCNA mutants for the five target partners (see Figure 2) using Y2H and SPR assays. Growth of the Y2H strain containing WT and mutant PCNA and the different partners was analyzed on selective agar plates lacking histidine. Growth rates similar to that of cells expressing WT PCNA are denoted as 1. Increase and decrease in affinity detected by Y2H or SPR assays are marked in bold.

c Fold increase or decrease in the PCNA-partner interaction affinities measured as the generation time of the Y2H strains grown in liquid selective media lacking histidine, relative to cells expressing WT PCNA (see Figures S4 and Materials and Methods).

d Fold increase in binding affinities of PCNA mutants for PIP peptides derived from the different partners (in parenthesis), relative to WT PCNA, as measured by SPR analysis. Binding affinities of the WT PCNA for Rad30, Pol32, Msh6, and Ung1 PIP peptides are 1.7×10^−7^M, 3.6×10^−7^M, 3×10^−6^M, and 1.2×10^−6^M, respectively. The off-rate (k_d_) of the Rad30 PIP peptide measured for the WT and pol30-Rad30E9 mutant are 0.03 s^−1^ and 0.00321 s^−1^, respectively.

e The spontaneous mutation rate was determined by fluctuation analysis (see Materials and Methods). Values in brackets represent the low and high limits for the 95% confidence interval obtained for each rate. The numbers in parentheses indicate the fold increase, as compared to the WT *POL30* strain. The differences between the mutation rate of the mutants and the WT are significant (*p*<0.002 in all cases, Mann-Whitney test).

f Strains containing these mutants as the sole source of PCNA are non-viable.

ND, not determined; NA, not applicable.

To verify that the selected PCNA mutants showing increased affinity for the PIP peptides also exhibit increased affinity for the full-length partner, a Y2H analysis was performed. For the Y2H assay, we used the YRG2 host strain, shown to be highly efficient in coupling the strength of protein-protein interactions with growth on media lacking histidine [32]. Selected PCNA mutants were characterized for their ability to bind each of 12 main PCNA partners [8] to obtain specificity profiles (Table 1, Table S1, and Figures S4–S5). Such profiling indicated, in most cases, that an increase in binding affinity for the target partner did not result in increased or decreased affinity for the other partners (Table 1, Table S1). These results demonstrate the high flexibility of the IDCL in terms of increased binding specificity. However, in some cases a strong trade-off between the bindings of different partners was observed. For example, some PCNA mutants selected for high affinity to Rad27 exhibited reduced affinity for Rad30 and vice versa (Table 1).

**Figure 4:**
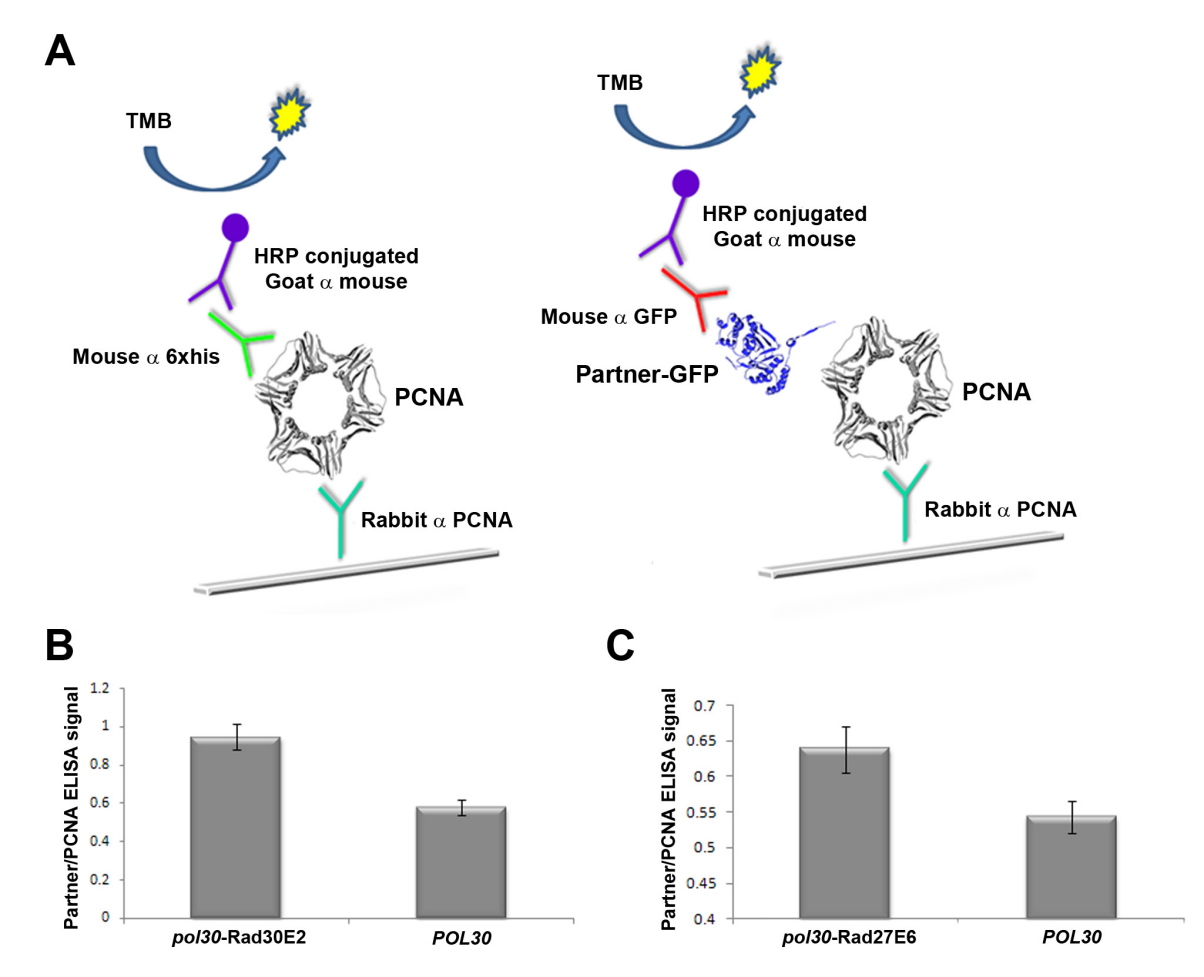
Increased levels of in vivo PCNA-partner complex formation in the *pol30*-Rad30E2 or the *pol30*-Rad27E6 mutant strains, relative to the *POL30* strain. (A). ELISA experimental set-up for the detection of PCNA-partner complexes extracted directly from WT and mutant PCNA strains. The assay is based on the immobilization of PCNA-partner complex using anti-PCNA antibody-coated ELISA plates. Detection of complex levels is performed using antibodies against the GFP fused to the C-terminal of the PCNA partner. The level of extracted complex is normalized to the total levels of PCNA immobilized on the plate using antibodies against a 6×histidine tag fused to PCNA. (B,C) The ELISA signal of the PCNA-Rad30 (B) or PCNA-Rad27 (C) complex normalized to the overall immobilized PCNA signal. The increase in complex formation in the *pol30*-Rad30E2 (B) and *pol30*-Rad27E6 (C) mutant strains, relative to the *POL30* WT strain, is 70% and 17% for the PCNA-Rad30 and PCNA-Rad27 complexes, respectively.

To examine whether increased PCNA-partner interaction affinities result in increased PCNA-partner complex formation in vivo in *pol30* mutant strains, we analyzed the levels of these complexes extracted from yeast cells. We used strains expressing the *pol30* mutants as a sole source of PCNA in the cell under the control of the native *POL30* promoter (see below). We immobilized PCNA from the crude yeast extracts onto ELISA plates and analyzed the amount of bound partner, relative to the amount of total PCNA immobilized on the plate (Figure 4). Using this approach, we successfully identified an increase of 70% and 17% in the amount of PCNA-Rad30 and PCNA-Rad27 complexes extracted from *pol30* mutant strains with increased affinity for Rad30 and Rad27, respectively, relative to the *POL30* strain (Figure 4). However, we did not detect an increase in PCNA-Msh6 or PCNA-Ung1 complex formation, probably due to the relatively minor increase in affinities of these interactions (1.6- and 3.5-fold, respectively, see Table 1) and the transient nature of PCNA-partner interactions [33].

To quantify differences in binding affinities of the PCNA mutants relative to the WT, we characterized four PCNA mutants with increased affinity for PIP peptides derived from Rad30, Pol32, Msh6, and Ung1 partners using SPR. These mutants were overexpressed in *E. coli*, purified by affinity chromatography, and immobilized on an SPR sensor chip for binding characterization [14]. SPR binding analysis enabled direct and sensitive measurement of interaction affinities relative to the Y2H system and indicated an up to 8-fold increase in binding affinity for the different PIP peptides, relative to WT PCNA (Table 1). Such analysis allowed the detection of a significant decrease in the Rad30 PIP peptide dissociation rate in the Pol30-Rad30E9p mutant relative to the WT, indicating a ∼9-fold increase in the lifetime of the PCNA-Rad30 complex (see Table 1). Collectively the ELISA, Y2H, and SPR assays validated and quantified the increase in mutant PCNA binding affinities, relative to WT PCNA.

To validate that the PCNA mutations have not altered the ability of that mutant to form an intact PCNA structure, we characterized six different PCNA mutants showing increased affinity for different partners. We used gel filtration chromatography analysis of the purified PCNA mutants to examine the molecular mass of the proteins under non-denaturating conditions. Using this approach, we found that all mutants form intact trimers of molecular mass of ∼90 kDa, similar to WT PCNA (Figure S6). As a control, we analyzed purified PCNA-52 mutant, which was previously shown to be defective in trimer formation [34], and detected a molecular mass of a monomer of ∼30 kDa (Figure S6). In addition, we examined the secondary structure content of the PCNA mutants, in comparison to the WT, using Circular Dichroism (CD) spectroscopy. This experimental approach allows examining whether the mutations in PCNA led to substantial structural alterations. We found that the CD spectra of the mutants are very similar to the CD spectrum of the WT PCNA (see 6). Overall, these results indicate that the PCNA mutations did not significantly alter the secondary structure of PCNA or its ability to form trimers.

Sequence analysis of the selected mutants revealed the presence of 3–4 mutations in the IDCL region out of the 4 positions randomized in the naïve library (Table 1). Surprisingly, some of the PCNA mutants identified following Rad27 PIP selection were characterized by a deletion of two amino acids after the first aspartic acid of the IDCL region (Figure 1B, Table 1) [25]. This deletion results in altered pattern of PCNA-partner specificity (Table 1) due to shortening of the exposed and flexible IDCL loop that may result in new conformational diversity. To validate the effect of the deletion, we generated the same deletion on the background of WT PCNA and observed similar binding specificity, relative to the selected deletion mutants (unpublished data).

### In Vivo Characterization of PCNA Mutants

**Figure 5:**
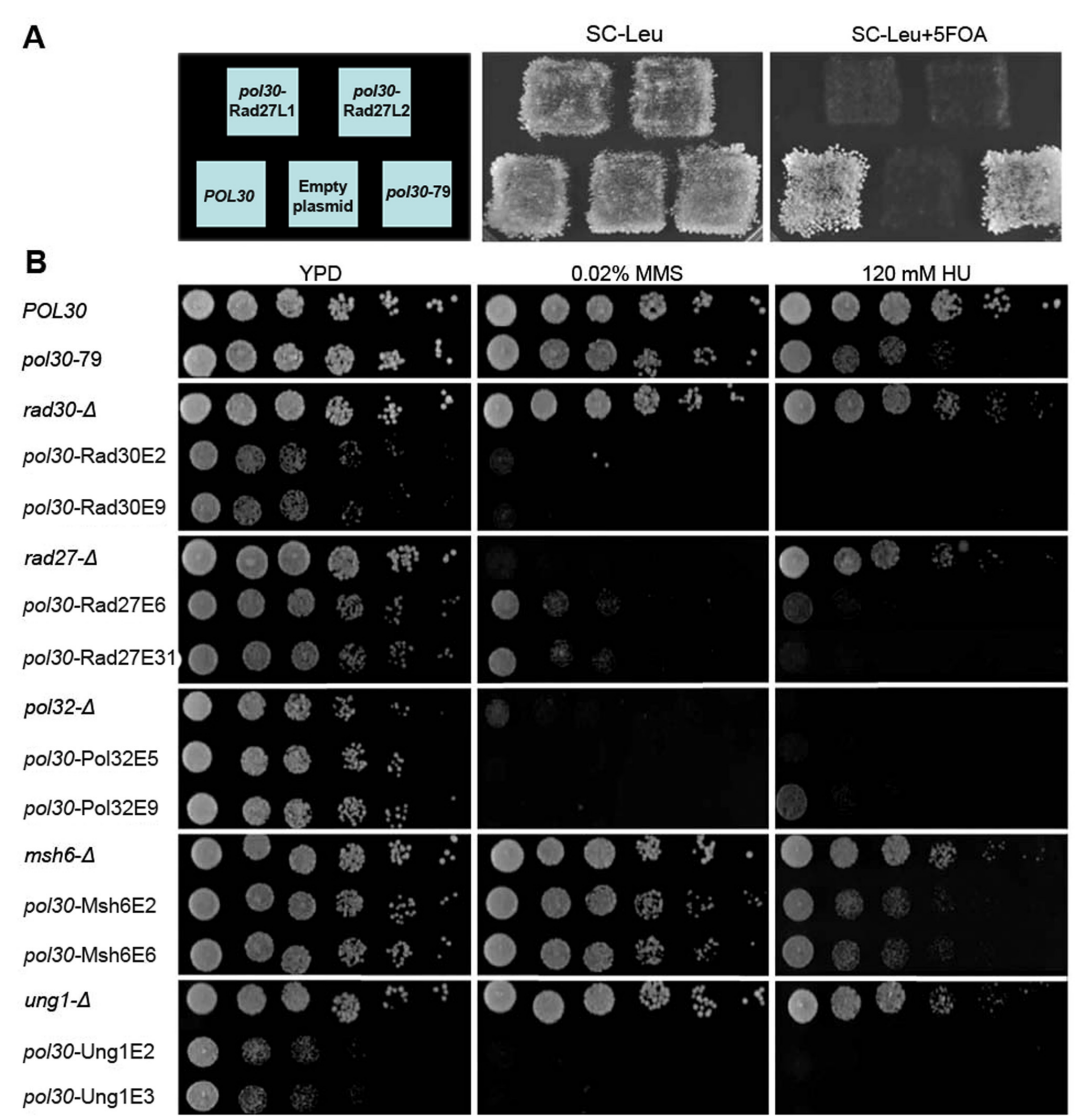
In vivo phenotypic analysis of PCNA mutants. (A) Non-viable strains containing PCNA mutants with increased binding affinity for Rad27 as the sole version of PCNA. Yeast strains containing WT PCNA expressed from a *URA3* plasmid and mutant PCNA genes expressed from a *LEU2* plasmid are viable (middle). Upon selection for loss of WT PCNA using 5-floororotic acid (5FOA), PCNA mutant strains are non-viable (right). (B) MMS (middle) and HU (right) sensitivity of strains containing PCNA mutants with increased binding affinity for Rad27, Pol32, Rad30, Msh6, and Ung1 and strains containing WT PCNA in which the same partners were deleted. For binding characterization and sequences of the mutants, see Table 1.

To study the in vivo ability of PCNA mutants to promote DNA replication and repair in yeast, we adopted a plasmid shuffling method to create haploid yeast strains carrying each mutant as the sole source of PCNA (see Materials and Methods). This approach allowed us to identify PCNA mutants leading to cell death, indicating their inability to support essential DNA replication processes (Figure 5A). In addition, many of the mutant strains exhibited high sensitivity to DNA damaging agents, such as hydroxyurea (HU) or methyl methanesulfonate (MMS), drugs that cause global replication stress and DNA alkylation, respectively (Figure 5B). Interestingly, the lethality or strong sensitivity displayed by our mutants presenting increased affinity for various partners far exceeds the sensitivity observed for the previously studied *pol30*-79 mutant, exhibiting a significant decrease in binding affinity for the majority of PCNA partners (Figure 5B) [31]. To verify that the replication defects in the mutant strains (Figure 5) are not due to reduced expression levels of the PCNA proteins, we confirmed the expression level of PCNA using western blot analysis and observed similar expression levels of all PCNA mutants, relative to WT PCNA (Figure S7).

**Figure 6:**
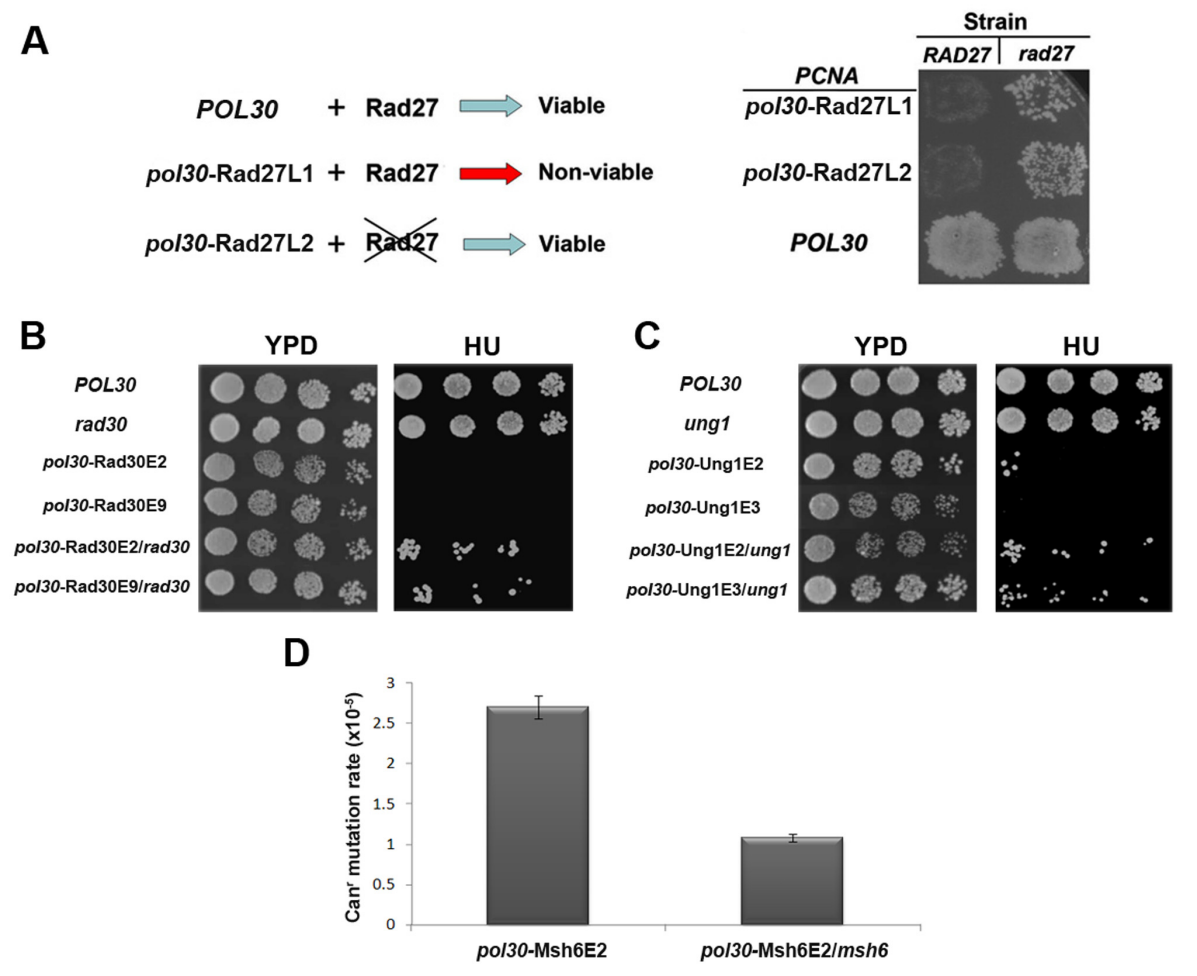
The suppression of *pol30* mutant strain phenotypes by the deletion of PCNA partner-encoding genes. (A) Lethality in yeast strains containing PCNA mutants with increased affinity for Rad27 can be suppressed by *rad27* deletion. General scheme describing the suppression of lethality (left). The growth analysis of *pol30* mutant and WT strains generated on the background of the parent or *rad27*-deleted strain (right). Strains containing each PCNA mutant as a sole source of PCNA were generated by transformation with a *URA3*-marked plasmid containing the *POL30* gene and a *LEU2*-marked plasmid containing *pol30*-Rad27L1, *pol30*-Rad27L2, or *POL30* (used as a control), followed by plating onto 5-FOA to force the loss of the *URA3* plasmid (see Materials and Methods for detailed description). (B,C) The suppression of *pol30*-Rad30E2, *pol30*-Rad30E9 (B), *pol30*-Ung1E2, and *pol30*-Ung1E3 (C) strain growth sensitivity phenotypes by *rad30* (B) or *ung1* deletion (C). *POL30* and the *pol30* mutant strains were generated and examined as described above. (D) Suppression of the level of spontaneous mutation rate of the *pol30*-Msh6E2 mutant strain by *msh6* deletion.

Next, we examined the sensitivity of strains containing single deletions of each of the five different partners on a WT PCNA background. We found that these strains exhibit equal or lesser phenotypic defects, relative to the selected PCNA mutant strains with increased affinity for the respective partner (Figure 5B). These results suggest that the cost for increasing PCNA-partner interaction affinity is equal to or much higher than the cost of that partner being absent (Figure 5B, see Rad30 and Ung1 as prominent examples). To further test this idea and validate that the mutations in PCNA do not disrupt any critical PCNA function unrelated to PCNA-partner interactions, we examined whether deletion of different partners on the background of the *pol30* mutant strains can suppress the growth sensitivity phenotypes. We first examined whether the *rad27* deletion can suppress the lethality of a strain containing the *pol30*-Rad27L1 or *pol30*-Rad27L2 mutants (Figure 6A). It was previously shown that *rad27* deletion does not cause lethality [35]. Interestingly, *rad27* deletion suppressed the lethality of the *pol30*-Rad27L1 and *pol30*-Rad27L2 strains probably due to the lack of PCNA-Rad27 complexes in these strains (Table 1, Figure 6A). This result demonstrates that the strong deleterious effect of PCNA mutants with increased binding affinity for Rad27 could be much higher than the effects of *rad27* deletion and firmly correlates with our in vitro and in vivo analyses of the PCNA mutants. We also found that the *rad27* deletion can suppress the phenotypes of other *pol30* mutant strains (Figure S8), however such suppression could be due to indirect pathway activation [36]. In addition, we examined whether *rad30* deletion, *ung1* deletion, and *msh6* deletion can suppress the phenotypes of the *pol30* mutants with increased affinity to Rad30, Ung1, and Msh6, respectively (Figure 6B–D). We found that indeed such deletions suppressed the growth sensitivity phenotypes of the *pol30* mutants with increased affinity for Rad30 or Ung1 and the high spontaneous mutation rate observed in the *pol30* mutant with increased affinity to Msh6 (Figure 6B–D). However, since the suppression of the strain phenotypes was not complete, additional factors, such as minor effects of the mutations on other PCNA-partner interactions, may play a partial role in the sensitivity phenotype of the examined *pol30* mutants. Collectively the suppression of the phenotypes of the *pol30* mutant strains by PCNA partner-deletions suggests that these phenotypes arise mainly as a result of specific enhancement of PCNA-partner interactions and that the *pol30* mutants are not defective in any critical function unrelated to PCNA-partner interactions. We also examined whether overexpression of different partners can suppress the growth sensitivity of the *pol30* mutants, however no suppression was observed (Figure S9).

**Figure 7:**
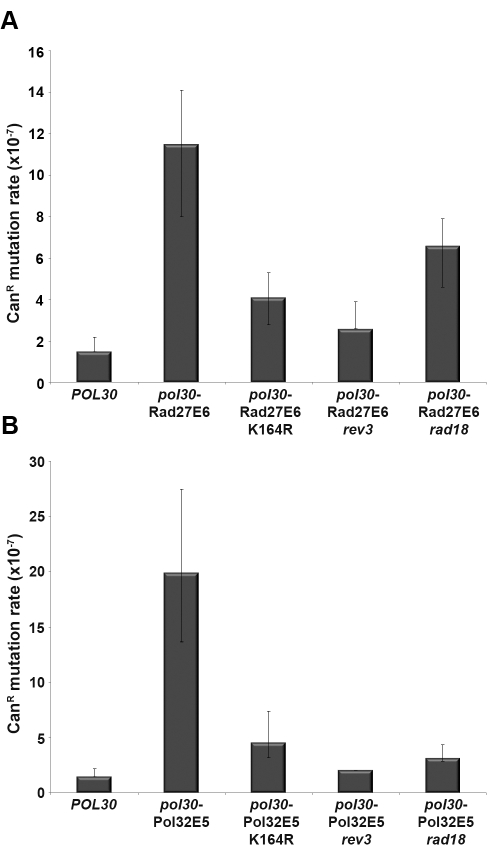
The increase in the spontaneous mutation rate of PCNA mutants is dependent upon Polζ recruitment and ubiquitination of PCNA at K164. The effects of the *rev3*, *rad18*, and K164R PCNA mutations on the spontaneous mutator phenotype of PCNA mutants with increased affinity for Rad27 (A, *pol30*-Rad27E6) and Pol32 (B, *pol30*-Pol32E5) are shown. The data are rates of Can^r^ mutation and are medians representing values that are within the 95% confidence limits for at least 25 independent determinations. The exact values of the spontaneous mutation rate in the different strains are presented in Table 2.

It was previously shown that PCNA contains another site of regulation (i.e. K164) that is modulated by ubiquitination or SUMOylation (Figure 1B) and that is crucial for the recruitment of TLS polymerases [13],[37]. To test whether the K164 regulatory site is active in the PCNA mutant strains, we examined the spontaneous mutation rate in these strains (Table 1). Increase in spontaneous mutation rate can indicate the recruitment of TLS polymerases to PCNA through K164 ubiquitination [13],[37]. Alternatively, these mutants can indirectly affect the spontaneous mutation rate by reducing DNA replication processivity and causing replication fork stalling, thereby leading to TLS recruitment [37]. These mutants can also indirectly alter Pol-δ or Pol-ε proofreading leading to TLS recruitment [37]. We measured the spontaneous mutation rate using the *CAN1* reporter assay by monitoring the ability of the PCNA mutant strains to grow in the presence of canavanine, a toxic analogue of arginine [38]. Interestingly, we observed a significant increase in the mutation rate in most of the PCNA mutant strains, relative to WT PCNA (Table 1). To examine whether Polζ, the major TLS polymerase [13],[37], is recruited in vivo to PCNA mutants showing increased affinity for Rad27 and Pol32 (*pol30*-Rad27E6 and *pol30*-Pol32E5, Table 1) upon K164 ubiquitination, we measured the spontaneous mutation rates of these mutant strains on the background of either mutated *rev3* encoding the catalytic subunit of Polζ or mutated *rad18* encoding the K164 ubiquitin ligase or K164R mutation (Figure 7, Table 2). We observed a dramatic reduction in the mutation rates of all of these strains, indicating that the high mutation rate in the *pol30* mutant strains is a result of Polζ recruitment to the PCNA mutants through K164 ubiquitination (Figure 7, Table 2). Collectively, these results indicate the functional separation between IDCL and K164 ubiquitination in regulating partner binding to PCNA.

**Table 2:** Spontaneous mutation rate analysis at the *CAN1* locus.

Genotype	Mutation Rate^c^	Fold Increase Relative to WT
*POL30*	1.5×10^−7^ [1.5, 2.2]	1
*pol30*-Rad27E6	1.2×10^−6^ [8.0, 13.9]	7.7
*pol30*-Rad27E6/K164R^a^	4.1×10^−7^ [2.8, 5.3]	2.7
*pol30*-Rad27E6/rev3^b^	2.6×10^−7^ [2.6, 3.9]	1.7
*pol30*-Rad27E6/rad18^b^	6.6×10^−7^ [4.5, 7.9]	4.4
*pol30*-Pol32E5	2.0×10^−6^ [13.6, 27.6]	13
*pol30*-Pol32E5/K164R^a^	4.6×10^−7^ [3.2, 7.4]	3.0
*pol30*-Pol32E5/rev3^b^	2.1×10^−7^ [2.1, 2.1]	1.4
*pol30*-Pol32E5/rad18^b^	3.2×10^−7^ [2.9, 4.5]	2.1

a Spontaneous mutation rate analysis of *pol30*-Rad27E6 or *pol30*-Pol32E5 mutant strains also containing the K164R mutation in the *POL30* gene.

b Spontaneous mutation rate analysis of *pol30*-Rad27E6 or *pol30*-Pol32E5 mutants generated on the background of the *rev3*- or *rad18*-deleted strains.

c The numbers in brackets represent the low and high values for the 95% confidence interval for each rate, obtained using the confidence interval for the median test. The medians and 95% confidence intervals were deduced from at least 25 independent determinations for each strain.

Finally, to examine the mutation spectra at the *CAN1* locus for pol30 mutant strains with increased affinity to Pol32 or Rad27, we sequenced the *CAN1* gene in individual canavanine resistance clones. We sequenced 37 and 26 *CAN1*-inactivating mutations isolated from the *pol30*-Pol32E5 and *pol30*-Rad27E6 mutants, respectively. We found that *pol30*-Pol32E5 mutant accumulated a broad range of mutations, including substitutions, frame-shifts, or deletions (Table S2). Interestingly, we observed that 20% of the mutations were characterized by a specific deletion of 67 nucleotides flanked by a 4 bp direct repeat (Table S2). This result suggests that polymerase slippage is one of the mutagenic mechanisms leading to the increased mutation rate of the *pol30*-Pol32E5 mutant strain [39]. In contrast, the *pol30*-Rad27E6 accumulated a very low frequency of deletions (4%) but a high rate of substitutions and frame-shifts (Table S2). We also analyzed the *CAN1*-inactivation mutations accumulated in the *pol30*-Pol32E5 and *pol30*-Rad27E6 mutants generated on the background of *rev3*-deletion or *pol30*-K164R mutation. We found that these mutants accumulated low levels of substitution with no deletions or frame-shifts (Table S2), suggesting that the diverse mutation spectra in the characterized *pol30* mutant strains is dependent upon *REV3* and K164 modification.

## Discussion

In this study, we have generated and examined a novel collection of PCNA mutants with increased binding affinity for several partners, relative to WT PCNA. We have established an integrated approach that allows us (1) to generate PCNA mutants with increased affinity for different partners, (2) to perform binding characterization of the mutants for many different partners in order to profile changes in binding specificity, and (3) to perform detailed in vivo characterization of the mutants for the detection of defects in DNA replication and repair. The generation of PCNA mutants with increased affinity for five different partners revealed the high plasticity of PCNA for increases in partner interaction affinities, implying that the WT IDCL sequence naturally evolved to bind multiple partners with moderate affinity, rather than adopting higher binding affinities for specific partners. This property could be a selectable evolutionary trait designed to maintain the dynamic nature of PCNA-partner interactions and to facilitate partner switching on PCNA. The large number of mutations and the lack of conservation observed in the selected mutants (Table 1) suggests that binding affinity for the PIP region involves diverse contributions from many IDCL residues and that multiple solutions exist for increases in PCNA-partner binding affinities (Table 1).

In vivo analysis of the different mutants revealed severe phenotypic defects, ranging from non-viability to high sensitivity to DNA-damaging agents (Figure 5). In contrast, abolishment of PCNA-partner interactions by mutating conserved residues in PCNA [31] or in the PIP region of the different partners [14],[16] or even by deletion of different partners results, in most cases, in relatively minor phenotypic defects (Figure 5) [14],[16]. Collectively, these results highlight the fragility of DNA replication and repair resulting from increased PCNA-partner interaction affinity, in contrast to the robustness detected in the face of abolishment of the same interactions. Functional redundancy in PCNA partners could be the major mechanism facilitating the robustness of the PCNA-partner interaction network in coping with an abolishment of PCNA-partners interactions [5]. Indeed, several publications have indicated that the exonuclease activity of Polδ can substitute for the 5′flap endonuclease activity of Rad27 in processing Okazaki fragments, thereby preventing genome instability [17],[40].

What could be the mechanistic basis for the severe phenotypic defects observed in the PCNA mutant strains? PCNA mutants showing increased binding affinities for different partners may experience prolonged PCNA-partner associations, thus altering partner switching at the PCNA IDCL region. Indeed, SPR analysis of the *pol30*-Rad30E9p mutant (Table 1) indicates that the increase in the binding affinity of a given partner is due to a decrease in that partner's dissociation rate (k_off_ rate, see Table 1). The phenotypic defects in the *pol30* mutant strains (Figure 5) were detected in strains containing PCNA mutants with relatively minor increases in affinity for different partners. SPR analysis indicated an up to 8-fold increase in binding affinity of PCNA for the different partners, indicating the high impact of such alterations on DNA replication and repair processes in vivo. Such defects indicate that affinity-based competition between different partners is a crucial factor for the regulation of PCNA-partner interactions during different stages of DNA replication and repair. This, moreover, suggests that these are highly dynamic processes that require multiple partner binding and dissociation events [41]. In support of this hypothesis, different stages of DNA replication and repair, such as lagging strand replication, TLS, and BER, require the sequential binding of multiple PIP-containing partners to the IDCL [8]. However, it is important to note that the mutations in the IDCL region of the *pol30* mutants leading to increased affinity for various partners can still disrupt other important in vivo functions of PCNA. For example, such mutations can affect the PCNA in vivo localization or loading onto the DNA, thus contributing to the phenotypic defects observed in the *pol30* mutant strains. Future work analyzing these in vivo PCNA properties would contribute to the characterization of the PCNA mutants described in this study. In addition, future in vitro assays would allow further analysis of the effects of increasing PCNA-partner interactions on partner switching on PCNA during the different steps of DNA replication and repair.

One of the phenotypes of the PCNA mutant strains is a significant increase in the spontaneous mutation rate at the *CAN1* gene, indicating the recruitment of TLS polymerases to PCNA (Table 1). To obtain deeper mechanistic insight into such recruitment, we have shown that this phenotype is suppressed by the deletion of either *rev3* [42] or *rad18* or by the K164R mutation (Figure 7) [12]. These results indicate that the increase in PCNA binding affinity for Rad27 and Pol32 can trigger the recruitment of Polζ to the replication fork via K164 ubiquitination. Furthermore, our data indicate a functional separation between the IDCL and K164 regulatory sites and suggest that the recruitment of Polζ by K164 modification can provide a back-up mechanism by which to sustain replication in cases of regulation defects involving the IDCL region. Examination of the mutation spectra at the *CAN1* locus of *CAN1*- resistant clones isolated from the *pol30*-Pol32E5 and *pol30*-Rad27E6 mutant strains indicated the accumulation of a broad range of mutations and a significant difference in the mutation spectra between these strains (Table S2). These results indicate that increased PCNA-partner interactions can lead to different mutations, demonstrating different characteristics of each mutant (Table S2). In addition, the *pol30*-Rad27E6 accumulated mutations were different from the mutations previously found in the *rad27*-deleted strain [35]. The mutations in the *rad27*-deleted strain were characterized by a high frequency of duplications, indicating severe impairment of lagging strand replication [35]. This comparison suggests that *pol30*-Rad27E6 affects the regulation of lagging strand replication, rather than leading to complete abolishment of Rad27 enzymatic activity.

To facilitate the generation and examination of PCNA mutants with increased affinity for several partners, we have developed an integrated platform based on directed evolution and yeast genetic approaches. Currently, the most common manner of employing genetics for studying the robustness of cellular processes addresses the all-or-none effects generated by gene knock-outs (5). The integrated approach that we have developed allows for the generation of much more subtle and controlled perturbations to reveal new properties of the DNA replication system. This approach does not directly affect the expression level of the proteins or the catalytic activities of the different partners, thus allowing for dissection of the effects of subtle alterations in PCNA-partner binding affinities on the replication process. In future studies, the mutants generated in this study could prove useful in efforts aimed at obtaining mechanistic insight into PCNA-partner binding and dissociation events during DNA replication and repair processes.

In summary, using DNA replication and repair as a model system, we have shown that biological processes can be highly robust to one set of perturbations yet at the same time be highly fragile to completely different perturbations. Our data thus provide a more balanced view on the robustness of biological processes and reveal that similar to many man-made complex systems, these processes possess both properties of robustness and fragility. Finally, the approach developed in this study, which allows for the generation of a variety of minor perturbations in a protein-protein interaction network, can be applied to study the molecular basis, mechanism, and fragility of other networks promoting different biological processes, including signal transduction and gene transcription.

## Material and Methods

### Plasmids

For *E. coli* expression, WT PCNA and the different mutants were cloned into plasmid pET28 (Novagen) using the NdeI and XhoI sites to yield a 6×Histidine-tagged version of the protein. For YSD, WT PCNA was cloned into plasmid pCTCON [22] using the NheI and BamHI sites to generate plasmid pCTCON-PCNA. For in vivo testing of PCNA mutants, a 200 bp PCNA-promoter region and a 300 bp PCNA-terminator region were amplified from genomic DNA using the fr-pro and rev-pro and the fr-ter and rev-ter primers for the promoter and terminator regions, respectively (Table S3). These fragments were cloned into the pRS315 and pRS316 centromeric plasmids using NotI and SpeI or HindIII and XhoI sites, respectively, to generate the pRS315-proterm and pRS316-proterm plasmids. WT and mutant PCNA genes were amplified using fr-pRS/PCNA and rev-pRS/PCNA primers and cloned into pRS315-proterm and pRS316-proterm plasmids by homologous recombination. PCNA partners were GFP-tagged at their natural locus using a GFP-cassette as previously described [43]. For the Y2H assay, the pAD-GAL4 and pBD-GAL4 plasmids (Stratagene) were used to clone WT or PCNA mutants and the various partners, respectively (see Table 1, Tables S1 and Table S3 for a list of partners and oligonucleotides, respectively).

### YSD of PCNA

PCNA was displayed on the yeast cell surface of EBY100 strain cells (see [22] for genotype) and analyzed by flow cytometry, essentially as described [22]. Briefly, EBY100 transformed with plasmid pCTCON-PCNA were grown in SDCAA media to logarithmic phase and 2×10^6^ of cells were washed, resuspended in SGCAA induction media, and grown at 20°C with shaking for an additional 18 h. Induced cells (1×10^6^) were collected by centrifugation, washed with PBSF (PBS+ 1 g/L BSA), and incubated for 1 h at 25°C with mouse α-Myc antibodies (Santa Cruz Biotechnology, 1 µl/50 µl PBSF) and 100–400 µM of biotinylated PIP peptide (Peptron, see Figure S1 for peptide sequences). Subsequently, cells were washed and incubated with FITC-conjugated α-mouse IgG (Sigma, 1 µl/50 µl PBSF) and APC-conjugated streptavidin (Jackson Immunoresearch, 1 µl/50 µl PBSF) for an additional hour on ice, with frequent mixing. The labeled cells were washed, resuspended with PBSF, and analyzed by flow cytometry (FACS Calibur, BD).

### Library Generation

Positions I121, A123, F125, and E129 of the IDCL region were fully randomized by two fragments overlapping PCR using plasmid pCTCON-PCNA as template. The two PCNA gene fragments were amplified using two sets of primers (fr-Lib1 and rev-NNS; rev-Lib1 and fr-NNS, Table S3), assembled, and further amplified using nested primers. A naïve library was generated by in vivo recombination to obtain ∼1×10^6^ colonies oversampling the PCNA library diversity.

### Library Selection

The naïve library was induced and labeled with different PIP peptides, as described above. EBY100 cells (1×10^7^) displaying the PCNA library were labeled, analyzed, and sorted using a FACS (Vantage, BD). Three to five iterative rounds of enrichment were performed. In each round, multiple “positive” events (3–5×10^4^), corresponding to cells found within the top 1%–2% of the green and red fluorescence intensity area, were collected into growth media and plated on agar plates for a new round of enrichment. For initial sorting of the naïve library, a sort gate of the top 5% of fluorescent cells was used. To increase the stringency of selection, a decreased peptide concentration was used in each subsequent round. Selection rounds were continued until no further enrichment was obtained.

### Cloning and Bacterial Expression of PCNA Mutants from FACS-Enriched Libraries

A pool of plasmids from the last round of FACS enrichment was PCR-amplified using the primers fr-pET/PCNA and rev-pET/PCNA (Table S3) and cloned into plasmid pET28. Single *E. coli* BL21 cells, transformed with the resulting plasmids, were inoculated into 10 ml LB media containing 50 µg/ml kanamycin, grown to OD_600_ 0.6, and induced with 1 mM of IPTG (Calbiochem) for 5 h at 30°C. The cells were then harvested, lysed in PBS supplemented with 0.1% Triton and 200 µg/ml lysozyme, centrifuged, and the cleared supernatant was collected. Total protein concentration of the different mutants was determined using a BCA protein assay kit (Thermo Scientific) and analyzed by SDS-PAGE to verify the similarity of PCNA expression levels.

### ELISA Screening of PCNA Mutants

ELISA plates (Griener Microlon 96W) were coated with 0.2 µg/ml streptavidin (Pierce) and 0.1 µg/ml of biotinylated PIP peptides, as described [44]. Following peptide coating, the plates were incubated with the cleared lysate generated above at appropriate dilutions and shaken at 25°C for 1 h. Plates were then washed with PBS supplemented with 0.05% Tween-80 (PBST) and each well was incubated with mouse α-6×His-tag antibodies (Santa-Cruz Biotechnology) diluted by a factor of 1∶2000 and then with secondary HRP-conjugated goat α-mouse antibodies (Jackson, 1∶5000). The HRP chromogenic TMB substrate solution (Dako) was added and the reaction was stopped by the addition of 100 µL of 1 M sulfuric acid and recorded at 450 nm using a Tecan Infinite M200 plate reader.

### Large-Scale Expression and Purification of WT and Mutant PCNA

*E. coli* BL21 cells were induced and lysed as above in a volume of 0.5 L with minor modifications. Briefly, following centrifugation the cell pellet was sonicated in 20 ml of lysis buffer, centrifuged, and the cleared supernatant was loaded on a pre-equilibrated column containing 2 mL Ni-NTA resin (Qiagen). The columns containing the lysates were gently shaken by inversion for 30 min at 25°C. The resin was then washed with 30 ml of wash buffer and PCNA was eluted in 1 ml fractions upon addition of elution buffer. Fractions containing PCNA were pooled and dialyzed against storage buffer. Protein concentration was determined with a BCA protein assay kit (Pierce) and analyzed by SDS-PAGE. The protein solutions were stored in 1 ml aliquots of 2 mg/ml at −20°C. Lysis, wash, elution, and storage buffers were derived from the activity buffer based on 300 mM NaCl, 50 mM Tris-HCl, pH 8, and supplemented with imidazole, according to the manufacturer's recommendations.

### Gel Filtration and CD Analysis of PCNA

WT and mutant PCNA were purified as described above. Gel filtration chromatography was performed on a Superdex 200 10/300 GL column (GE Healthcare) using the ÄKTApurifier FPLC system. All proteins were run in activity buffer at monomer concentrations of 3–8 µM at which WT PCNA is a trimer and Pol30-52 is a monomer [34]. CD spectra were obtained for WT PCNA and six representative mutants using the Jasco J-810 CD Spectropolarimeter. All measurements were performed at room temperature in activity buffer. Data were obtained for the wavelength range of 204–260 nm and normalized to protein concentration to obtain molar ellipticity.

### SPR Analysis of Selected Mutants

Protein interaction assays were carried out using the ProteOn XPR36 (Bio-Rad) instrument. WT or PCNA mutants (0.4 to 5.2 fmol) were immobilized on the surface of a GLM sensor chip by a carbodiimide-activated succinimide-coupling method, as specified by the manufacturer. All SPR experiments were performed by flowing 150 µl of the target peptide at a flow rate of 30 µl/min onto the PCNA-bound chip. Different concentrations (5–5,000 nM) of PIP peptides (Table 1) were injected over the PCNA chip, and binding parameters were determined using ProteOn XPR36 software (Bio-Rad). The ligand (PCNA) and analyte (peptide) buffers were PBST and 150 mM NaCl, 1 mM EDTA, 0.01% Tween-80, 30 mM Hepes, pH 7.5, respectively.

### Y2H Analysis

Y2H analysis was performed using the Yeast Two Hybrid Phagemid vector kit (Stratagene), following the manufacturer's instructions. The pAD-PCNA-WT/mutant plasmids were used as bait while plasmids encoding 12 different PCNA partners (Table 1 and Table S1) were used as prey. The YRG2 host strain (Stratagene) was cotransformed with pAD-PCNA WT/mutant and pBD-partner plasmids in all possible combinations using the LiAC method. Single transformants were grown in liquid SC-Leu-Trp to O.D_600_ 10, washed twice with DDW, and diluted to an initial OD_600_ of 0.3. A series of 10-fold serial dilutions was then spotted onto selective SC-Leu-Trp-His plates and incubated at 30°C for 3 d. For quantitative Y2H, the generation time of the indicated mutants and their respective partners were calculated from their growth curves in liquid SC-Leu-Trp-His media. Cells were grown overnight in SC-Leu-Trp, washed twice with ddH2O, and diluted by a factor of 1∶50 into 10 ml of pre-warmed SC-Leu-Trp-His. O.D_600_ measurements of the cultures were taken at the indicated time; the generation time (τ) was calculated from the growth curves according to the equation OD_t_ = OD_0_×2^t/τ^. The generation time calculated for each culture is an average of at least 3 independent experiments.

### In Vivo Characterization of Novel Mutants Serving as the Sole PCNA Source

Novel haploids containing PCNA mutants were generated using the plasmid shuffling method. Briefly, a pol30::KanMX magic marker heterozygote diploid strain BY4743 (Open Biosystem) was transformed with plasmid pRS316-*POL30*. Following dissection of the diploid, a haploid containing the *CAN+* gene and plasmid pRS316-*POL30* as a sole source of PCNA was generated. This host strain was transformed with selected pRS315-*pol30* mutants, plated on SC-Ura-Leu plates, followed by replica plating to SC-Leu+5FOA plates. Haploids, containing PCNA mutants as a sole source, were further verified by plating on either SC-Leu or SC-Ura plates. For testing selected PCNA mutants, a haploid containing rad27::HYG, rad30::HYG, pol32::HYG, msh6::HYG, or ung1::HYG were generated using plasmid pAG32 by conventional genetic approaches. Growth of the PCNA mutant strains in the presence of 120 mM HU (Toronto Research Chemicals) or 0.02% MMS (Sigma) was performed as described [45]. To examine the effects of partner overexpression on pol30 mutant strains, these strains were transformed with various plasmids containing GST-tagged PCNA partner encoding genes under the control of a GAL1/10 inducible promoter, as previously described [46]. Overnight cultures were plated in serial dilutions on SC-Ura containing either glucose or galactose with or without DNA-damaging agents, as described above.

### Analysis of Spontaneous Mutation Rates and Mutation Spectra at the *CAN1* Gene

The mutation rates for the different *pol30* mutant strains described in this study were determined by fluctuation test analysis using the Lea and Coulson method [47],[48]. The different strains were plated as single colonies on SC-Leu plates and allowed to grow for 3 d at 30°C. At least 25 single colonies from each strain were excised from the plate and resuspended in 1 ml of sterile water to an O.D_600_ of 0.7. Appropriate dilutions of the cells were then plated on SC-Leu and SC-Leu-Arg+canavanine (60 mg/ml) to obtain the number of viable cells (Nt) and the number of canavanine-resistant cells (r), respectively. Using the Lea and Coulson method [47], the number of mutations (m) per colony was derived from the number of canavanine resistant-colonies (r) across parallel cultures, using the following equation: m/r-ln(m)−1.24 = 0. The m values were then used to calculate the mutation rate, M, using the following equation: M = m/Nt, where Nt is the average number of viable cells per plating. The different M values were sorted to obtain the median. The low and high values for the 95% confidence interval for each rate were obtained using the confidence interval median test. The m, M, and 95% confidence interval values were determined using the Fluctuation Analysis CalculatOR (FALCOR) program, with r and Nt as the input values (http://www.keshavsingh.org/protocols/FALCOR.html) [49]. The significance of differences between the mutation rates of the mutants and the WT was estimated by the Wilcoxon-Mann-Whitney test to obtain *p* values. To analyze the mutation spectra of *pol30* mutant strains, genomic DNA was extracted from individual *CAN1*-resistant colonies. The *CAN1* locus was PCR amplified using upstream and downstream primers and the PCR product was sequenced using 3 primers spanning the entire ORF. Analysis of sequences was performed using the Geneious program.

### ELISA for Detecting PCNA-Partner Complexes Extracted from Yeast Cells

Yeast cell extracts were generated from 0.5 L of logarithmic cultures using conventional methods. Briefly, cell pellets were lysed with Cell Lytic (Sigma), supplemented with protease inhibitors (Sigma) and glass beads, as suggested by the manufacturer. Following centrifugation, cell extracts were collected and protein concentration was determined by the BCA method. ELISA plates coated with rabbit α-PCNA antibodies (1∶3000, Adar Biotech) were incubated with 100 µl of yeast cell extract at a protein concentration of 3 mg/ml for 1 h at RT. Following 3 washing steps with PBST, wells were incubated with either mouse α-His antibodies (1∶500, Santa Cruz Biotechnology), to detect PCNA adsorption, or α-GFP antibodies (1∶500, Roche), to detect the presence of GFP-tagged PCNA partners bound to PCNA (see Figure 4). Plates were then washed 3 times with PBST and incubated with secondary HRP-conjugated goat α-mouse antibodies (1∶2000, Jackson). PCNA-partner complex levels were calculated as the ratio of the GFP signal to the PCNA signal detected for the same cell extract. Values represent averages of at least 5 independent repeats.

### Western Blot Analysis of Crude Yeast Extracts

Selected haploid PCNA mutants were grown to OD_600_ 0.8, centrifuged, and lyzed using cell lytic solution (Sigma) supplemented with protease inhibitor cocktail (Sigma), following the manufacturer's instructions. Following TCA treatment, samples containing 10 µg of crude lysates were loaded on a 10% SDS-PAGE gel and subjected to western blot analysis using rabbit α-PCNA (custom-made by Adar Biotech, 1∶2000 in PBS+1% BSA) and mouse α-Pgk1 (Invitrogen, 1∶7000 in PBS+1% BSA) antibodies. Antibody binding was detected using either HRP-conjugated goat α-rabbit (1∶10,000) or HRP-conjugated goat α-mouse (1∶10,000) antibodies, respectively. The latter were used to detect the yeast Pgk1 protein that served as a loading control.

## Supporting Information

Figure S1 **PIP peptide sequences derived from the different target partners (see text for detailed explanation).** Conserved residues are highlighted.
(0.50 MB TIF)

Figure S2 **Sequence alignment of the IDCL region of PCNA from different organisms.** The four non-conserved residues that were completely diversified in the mutant library are highlighted with a red arrow. The PCNA SUMOylation site was not mutated and is highlighted with a blue arrow.
(0.85 MB TIF)

Figure S3 **Yeast surface display of PCNA. (A) PCNA is displayed as an Aga2 (grey) fusion on the yeast cell surface.** Expression is detected through fluorescent antibody binding to the c-Myc epitope tag (light blue) while binding of the biotinylated PIP peptide (orange) is detected using fluorescently-labeled streptavidin (green). (B–D) Flow cytometry dot plots of yeast cells displaying WT PCNA (B–C) and the inactive PCNA79 mutant (D) incubated with fluorescein isothiocyanate (FITC)-labeled antibodies to the c-Myc epitope (*x*-axis) to analyze PCNA display levels. The specificity of PCNA binding for PIP peptides was detected following incubation with biotinylated PIP peptide derived from Rad30 (B and D) and mutated Rad30 in which the two conserved phenylalanine residues of the PIP peptide are mutated to alanine (C), followed by incubation with allophycocyanin (APC)-labeled streptavidin (*y*-axis).
(0.06 MB PDF)

Figure S4 **Yeast two hybrid analysis of selected PCNA-partner interactions.** The WT and mutant PCNA were fused to the DNA-activating domain (pAD) and the Rad30 (A), Rad27 (B), Pol32 (C), MSH6 (D), and UNG1 (E) partners were fused to the DNA-binding domain (pBD). The transformed YRG2 yeast strains were grown on liquid selective media lacking leucine (L), tryptophan (W), and histidine (H, right) to detect for PCNA-partner interactions.
(0.15 MB TIF)

Figure S5 **Yeast two hybrid analysis of selected PCNA-partner interactions.** The WT and mutant PCNA were fused to the DNA-activating domain (pAD) and the Rad30 (A), Rad27 (B), Pol32 (C), MSH6 (D), and UNG1 (E) partners were fused to the DNA-binding domain (pBD). The transformed YRG2 yeast strains were serial diluted and spotted on selective plates lacking leucine (L) and tryptophan (W, left) and then spotted on selective plates further lacking histidine (H, right) to detect for PCNA-partner interactions.
(0.30 MB TIF)

Figure S6 **Analysis of PCNA trimers and secondary structure contents of six PCNA mutants and WT PCNA.** (A) Gel filtration chromatography analysis of WT PCNA (Pol30p) and the six PCNA mutants indicate no significant alterations in trimer formation. The previously identified PCNA mutant defective in trimerization (Pol30-52, [34]) elutes as a peak at ∼16 ml, corresponding to the monomeric form of PCNA. The retention volumes and molecular masses (∼90 kDa for the trimer and 30 kDa for the monomer) were calibrated using standard molecular markers. (B) Circular dichroism analysis of PCNA WT and mutants, indicating similar secondary structure contents.
(0.65 MB TIF)

Figure S7 **Western blot analysis of the expression level of WT and selected PCNA mutants in yeast (upper panel): 1-** ***pol30*** **-Rad27E6, 2-** ***pol30*** **-Rad30E2, 3-WT PCNA, 4-** ***pol30*** **-79, 5-** ***pol30*** **-Pol32E5, 6-** ***pol30*** **-Ung1E2, 7-** ***pol30*** **-Msh6E2 (see** **Table 1** **in the main text for sequence and characterization of the mutants) using anti-PCNA antibodies.** The expression of Pgk1 was monitored as a loading control using anti-Pgk1 antibodies (bottom panel).
(0.03 MB PDF)

Figure S8 **WT and** ***pol30*** **mutant sensitivity examined on the background of the parent (left) or** ***rad27*** **-deleted (right) strains.** The *rad27* deletion results in suppression of the growth sensitivity in the case of the *pol30*-Rad30E2 or *pol30*-Ung1E2 strains. In contrast, the rad27 deletion had only weak effect on the growth sensitivity of the *pol30*-Rad27E6 or *pol30*-Msh6E2 mutant strains.
(0.28 MB DOC)

Figure S9 **The effects of overexpression of Rad27, Rad30, Pol32, and Msh6 on the growth sensitivity of the** ***POL30*** **,** ***pol30*** **-79,** ***pol30*** **-Rad27E6, or** ***pol30*** **-Pol32E5 strain.** Overexpression of the different partners did not reduce the growth sensitivity of the strains to DNA damaging agents.
(0.43 MB TIF)

Table S1 **Yeast two hybrid analysis of binding of selected PCNA mutants with seven additional partners not described in** **Table 1** **of the main text.**
(0.01 MB DOC)

Table S2 ***CAN1*** **mutation spectra for** ***POL30*** **mutants.**
(0.08 MB DOC)

Table S3 **Sequences of oligonucleotides used in this study.**
(0.05 MB DOC)

## Abbreviations

APC: allophycocyanin
BER: base excision repair
CD: Circular Dichroism
ELISA: enzyme-linked immunosorbent assay
FACS: fluorescence-activated cell sorting
HU: hydroxyurea
IDCL: inter-domain connecting loop
MMR: mismatch repair
MMS: methyl methanesulfonate
PCNA: proliferating cell nuclear antigen
PIP: PCNA-interacting protein
SPR: surface plasmon resonance
TLS: translesion synthesis
Y2H: yeast two hybrid
YSD: yeast surface display
