# Supplementary figures and images for: Subtle Alterations in PCNA-Partner Interactions Severely Impair DNA Replication and Repair
Source: PLoS Biol. 2010 Oct 12;8(10):e1000507. doi: 10.1371/journal.pbio.1000507 (PMC2953525; doi:10.1371/journal.pbio.1000507)

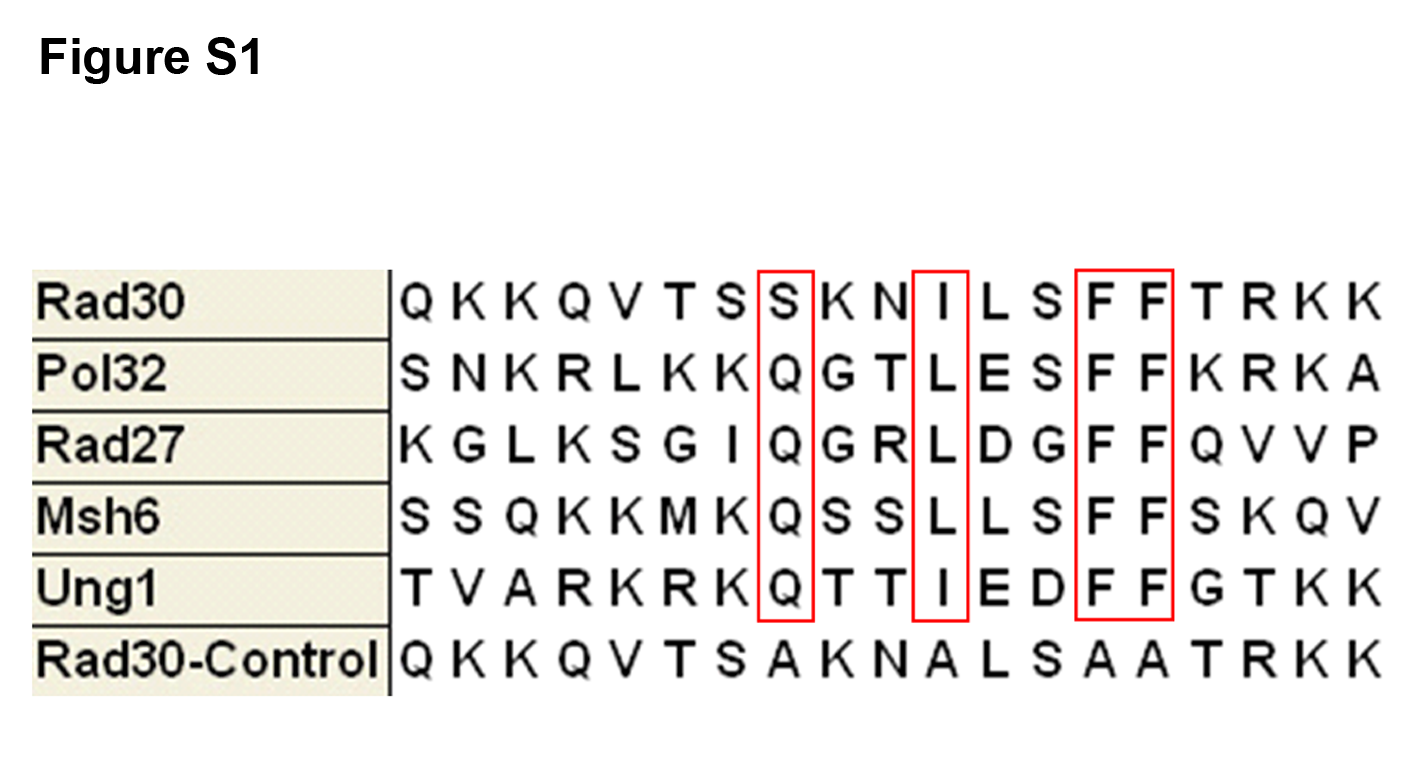

Supplement: Figure S1 — PIP peptide sequences derived from the different target partners (see text for detailed explanation). Conserved residues are highlighted. (0.50 MB TIF) [file pbio.1000507.s001.tif]

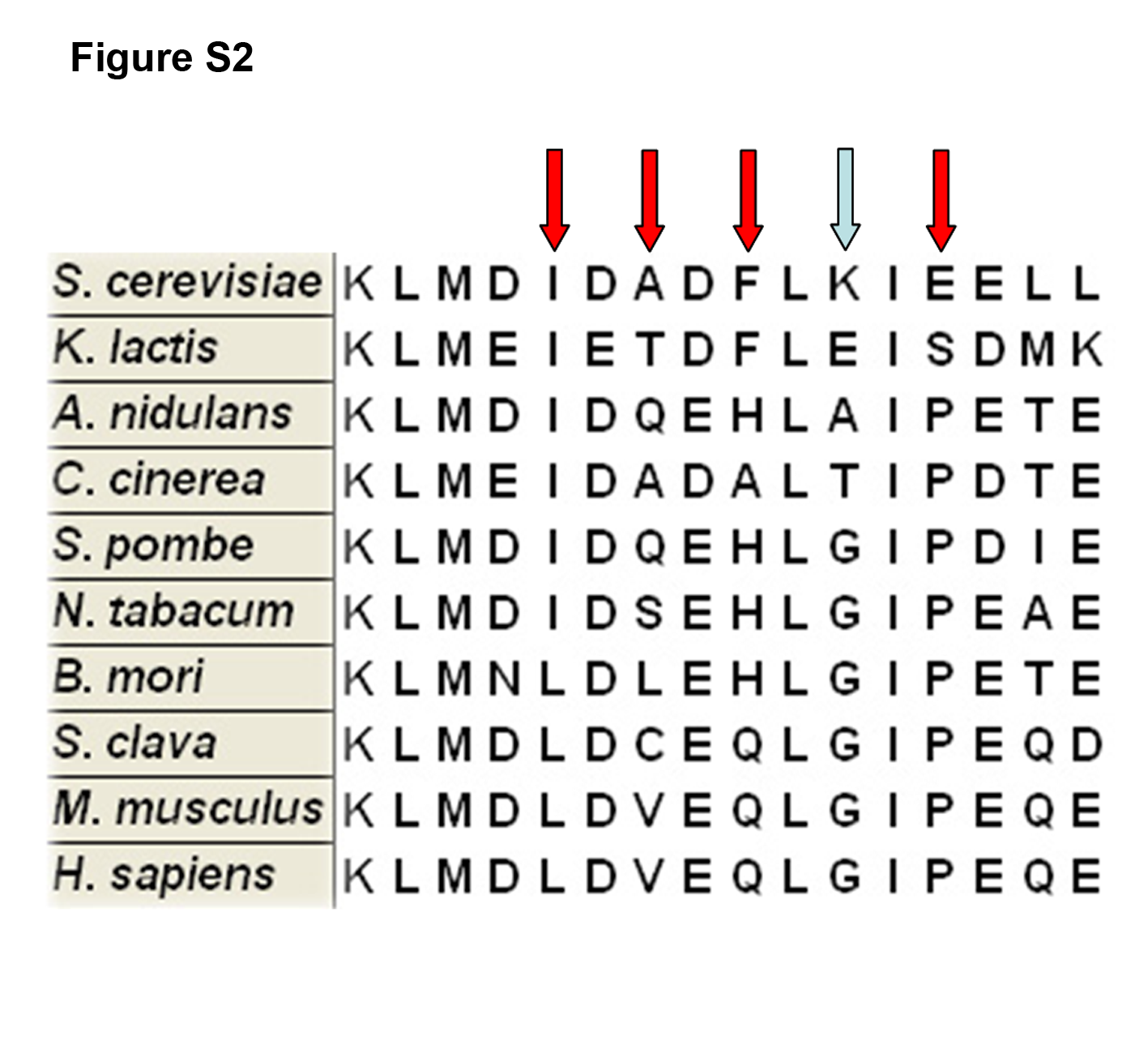

Supplement: Figure S2 — Sequence alignment of the IDCL region of PCNA from different organisms. The four non-conserved residues that were completely diversified in the mutant library are highlighted with a red arrow. The PCNA SUMOylation site was not mutated and is highlighted with a blue arrow. (0.85 MB TIF) [file pbio.1000507.s002.tif]

Figure S3

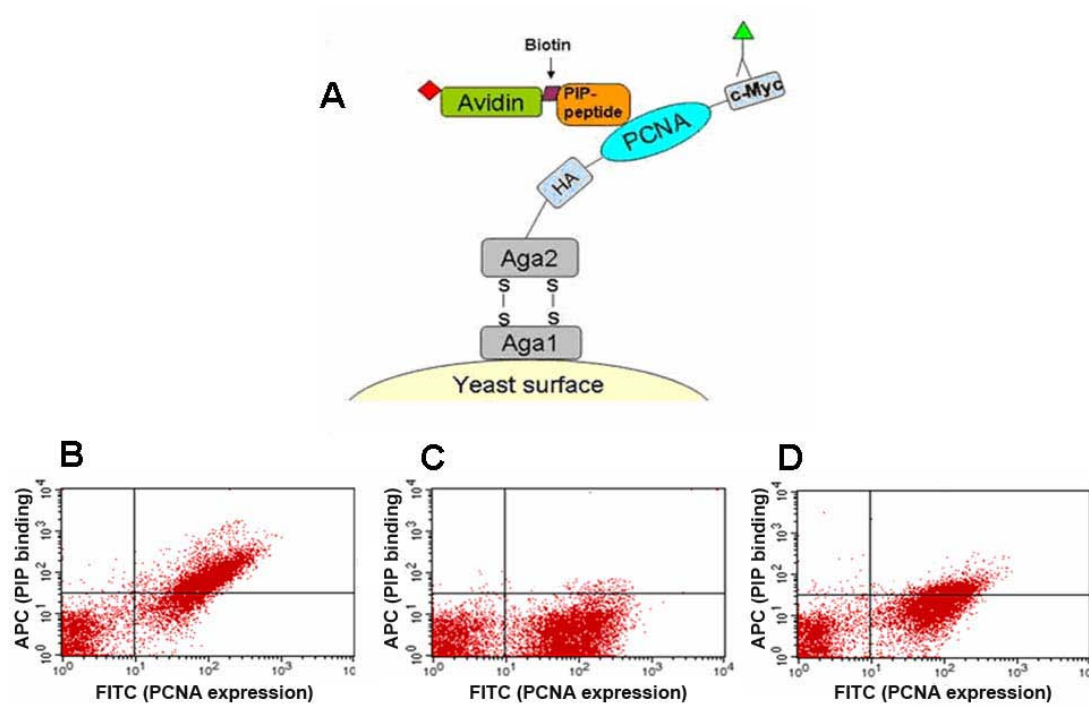

Supplement: Figure S3 — Yeast surface display of PCNA. (A) PCNA is displayed as an Aga2 (grey) fusion on the yeast cell surface. Expression is detected through fluorescent antibody binding to the c-Myc epitope tag (light blue) while binding of the biotinylated PIP peptide (orange) is detected using fluorescently-labeled streptavidin (green). (B–D) Flow cytometry dot plots of yeast cells displaying WT PCNA (B–C) and the inactive PCNA79 mutant (D) incubated with fluorescein isothiocyanate (FITC)-labeled antibodies to the c-Myc epitope (x-axis) to analyze PCNA display levels. The specificity of PCNA binding for PIP peptides was detected following incubation with biotinylated PIP peptide derived from Rad30 (B and D) and mutated Rad30 in which the two conserved phenylalanine residues of the PIP peptide are mutated to alanine (C), followed by incubation with allophycocyanin (APC)-labeled streptavidin (y-axis). (0.06 MB PDF) [file pbio.1000507.s003.pdf]

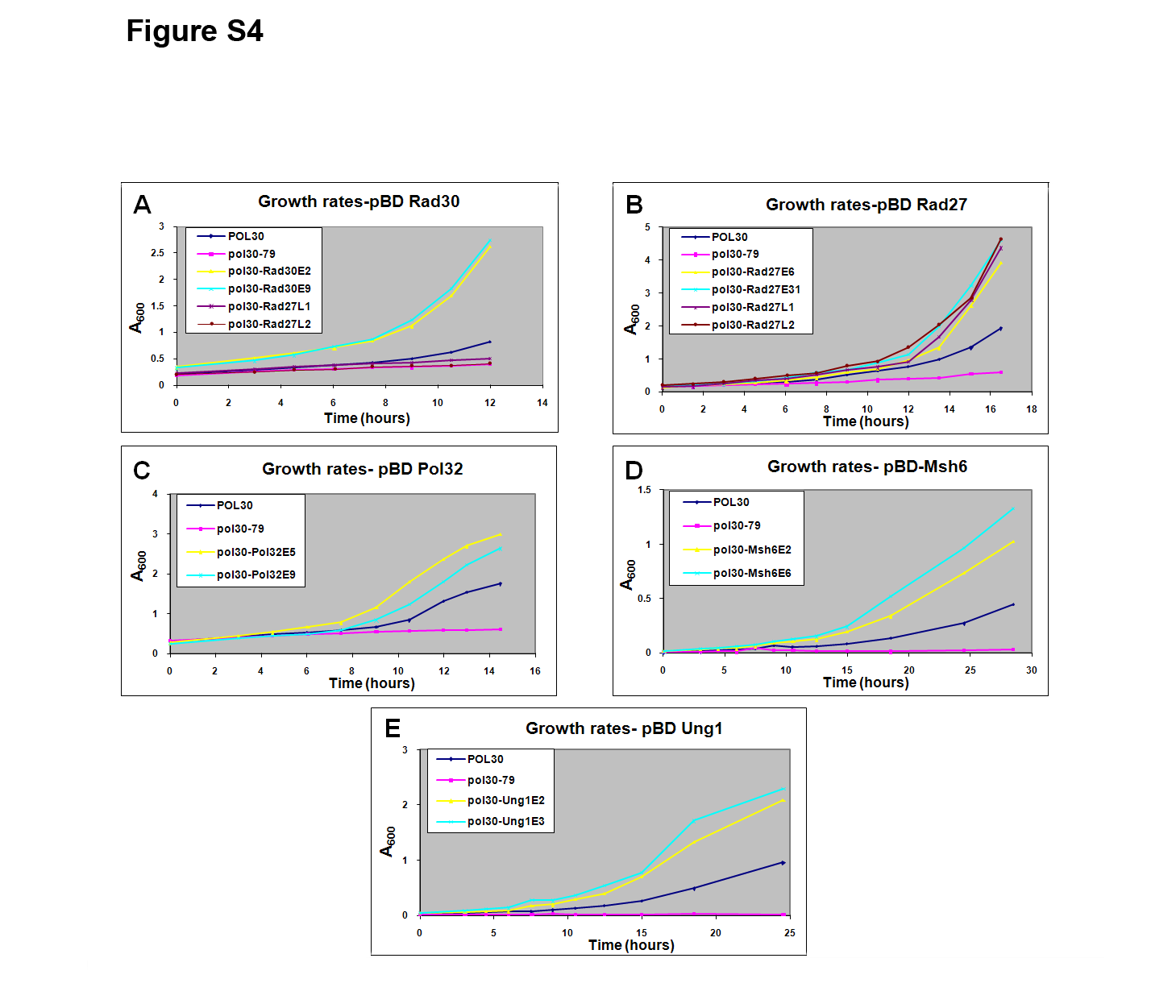

Supplement: Figure S4 — Yeast two hybrid analysis of selected PCNA-partner interactions. The WT and mutant PCNA were fused to the DNA-activating domain (pAD) and the Rad30 (A), Rad27 (B), Pol32 (C), MSH6 (D), and UNG1 (E) partners were fused to the DNA-binding domain (pBD). The transformed YRG2 yeast strains were grown on liquid selective media lacking leucine (L), tryptophan (W), and histidine (H, right) to detect for PCNA-partner interactions. (0.15 MB TIF) [file pbio.1000507.s004.tif]

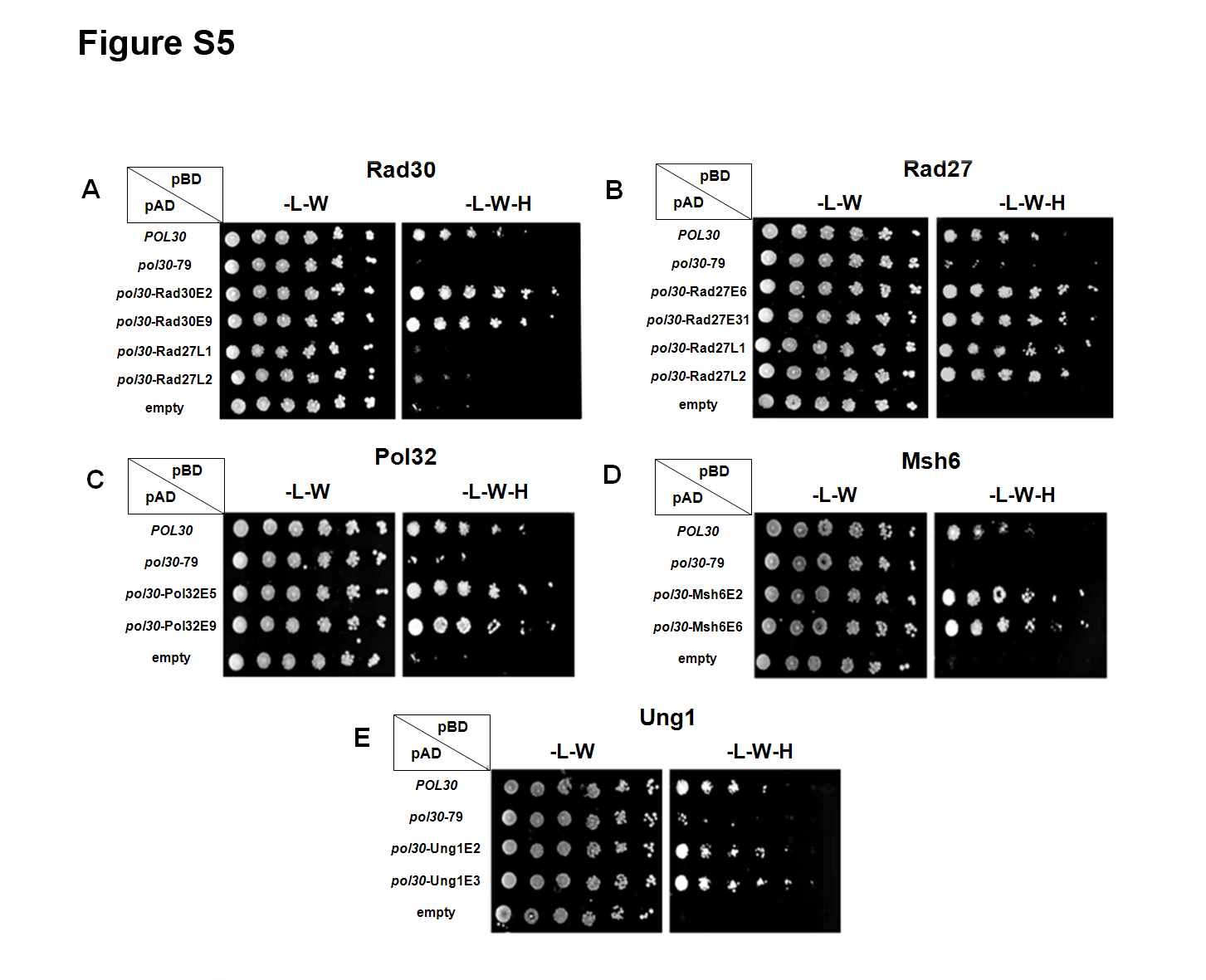

Supplement: Figure S5 — Yeast two hybrid analysis of selected PCNA-partner interactions. The WT and mutant PCNA were fused to the DNA-activating domain (pAD) and the Rad30 (A), Rad27 (B), Pol32 (C), MSH6 (D), and UNG1 (E) partners were fused to the DNA-binding domain (pBD). The transformed YRG2 yeast strains were serial diluted and spotted on selective plates lacking leucine (L) and tryptophan (W, left) and then spotted on selective plates further lacking histidine (H, right) to detect for PCNA-partner interactions. (0.30 MB TIF) [file pbio.1000507.s005.tif]

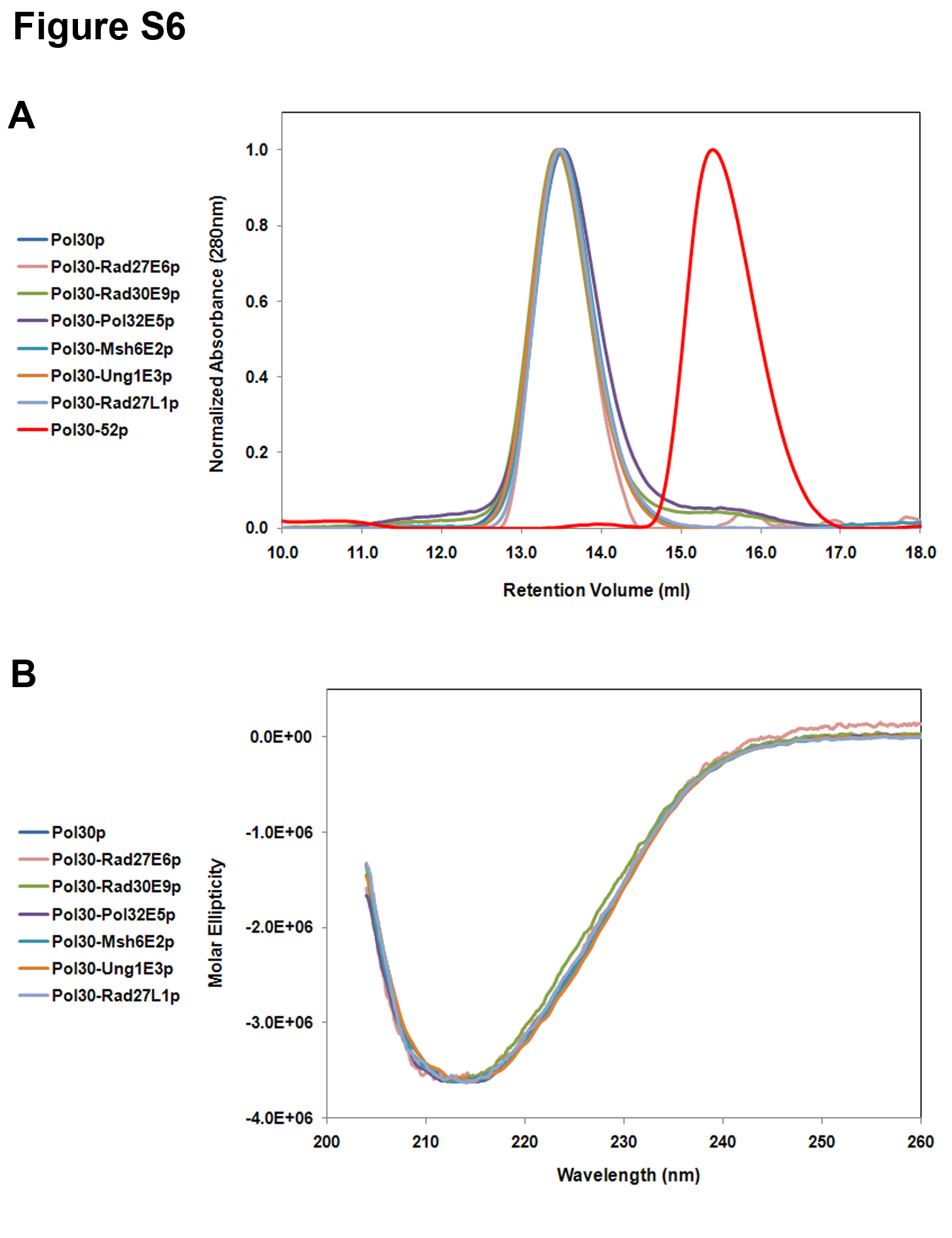

Supplement: Figure S6 — Analysis of PCNA trimers and secondary structure contents of six PCNA mutants and WT PCNA. (A) Gel filtration chromatography analysis of WT PCNA (Pol30p) and the six PCNA mutants indicate no significant alterations in trimer formation. The previously identified PCNA mutant defective in trimerization (Pol30-52, [34]) elutes as a peak at ∼16 ml, corresponding to the monomeric form of PCNA. The retention volumes and molecular masses (∼90 kDa for the trimer and 30 kDa for the monomer) were calibrated using standard molecular markers. (B) Circular dichroism analysis of PCNA WT and mutants, indicating similar secondary structure contents. (0.65 MB TIF) [file pbio.1000507.s006.tif]

Figure S7

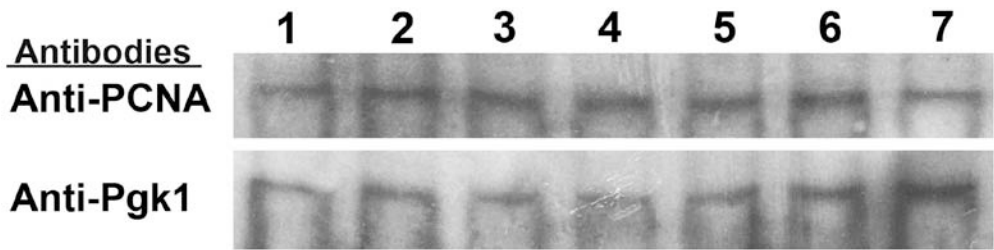

Supplement: Figure S7 — Western blot analysis of the expression level of WT and selected PCNA mutants in yeast (upper panel): 1- pol30 -Rad27E6, 2- pol30 -Rad30E2, 3-WT PCNA, 4- pol30 -79, 5- pol30 -Pol32E5, 6- pol30 -Ung1E2, 7- pol30 -Msh6E2 (see Table 1 in the main text for sequence and characterization of the mutants) using anti-PCNA antibodies. The expression of Pgk1 was monitored as a loading control using anti-Pgk1 antibodies (bottom panel). (0.03 MB PDF) [file pbio.1000507.s007.pdf]

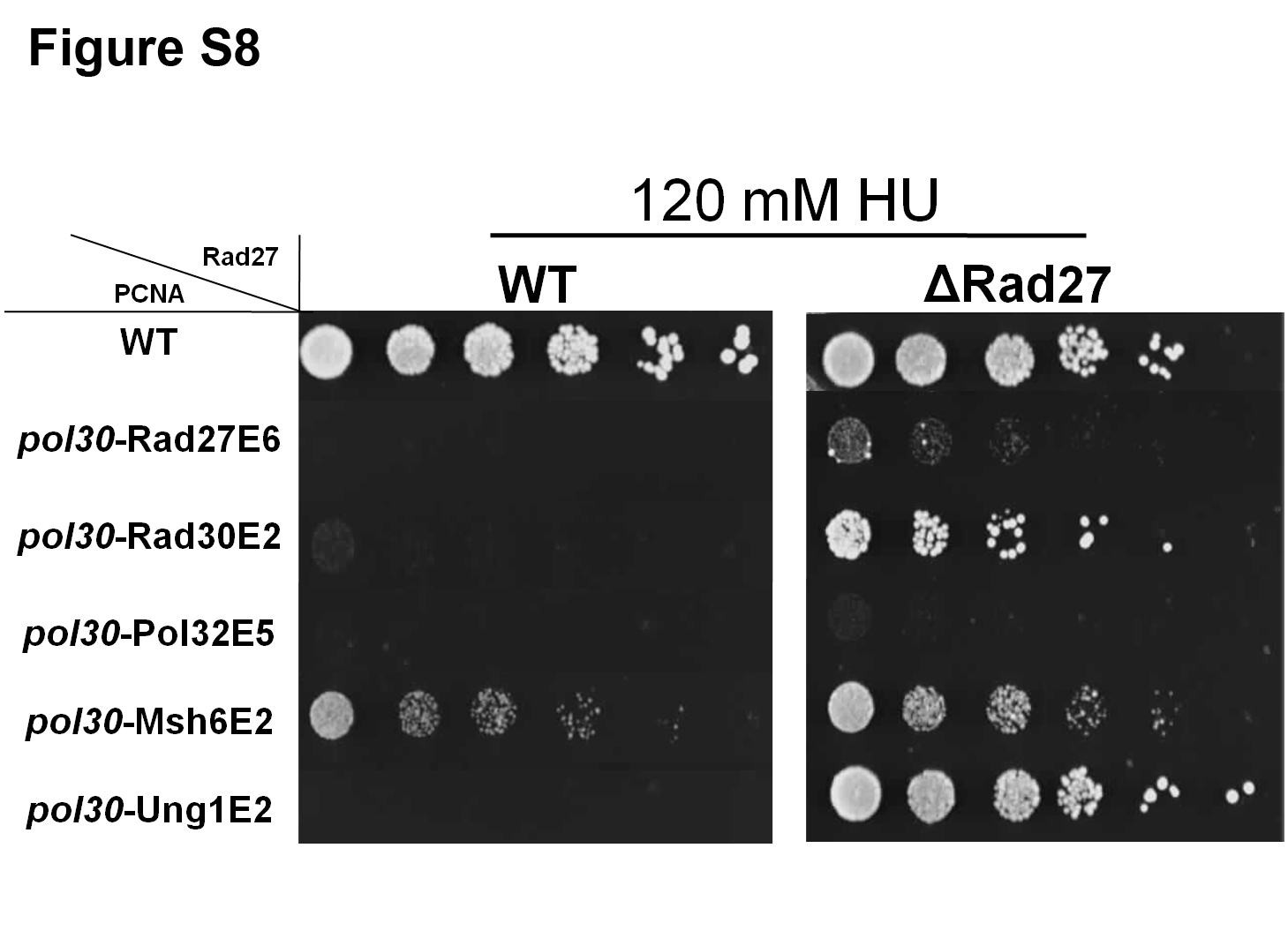

Supplement: Figure S8 — WT and pol30 mutant sensitivity examined on the background of the parent (left) or rad27 -deleted (right) strains. The rad27 deletion results in suppression of the growth sensitivity in the case of the pol30-Rad30E2 or pol30-Ung1E2 strains. In contrast, the rad27 deletion had only weak effect on the growth sensitivity of the pol30-Rad27E6 or pol30-Msh6E2 mutant strains. (0.28 MB DOC) [file pbio.1000507.s008.tif]

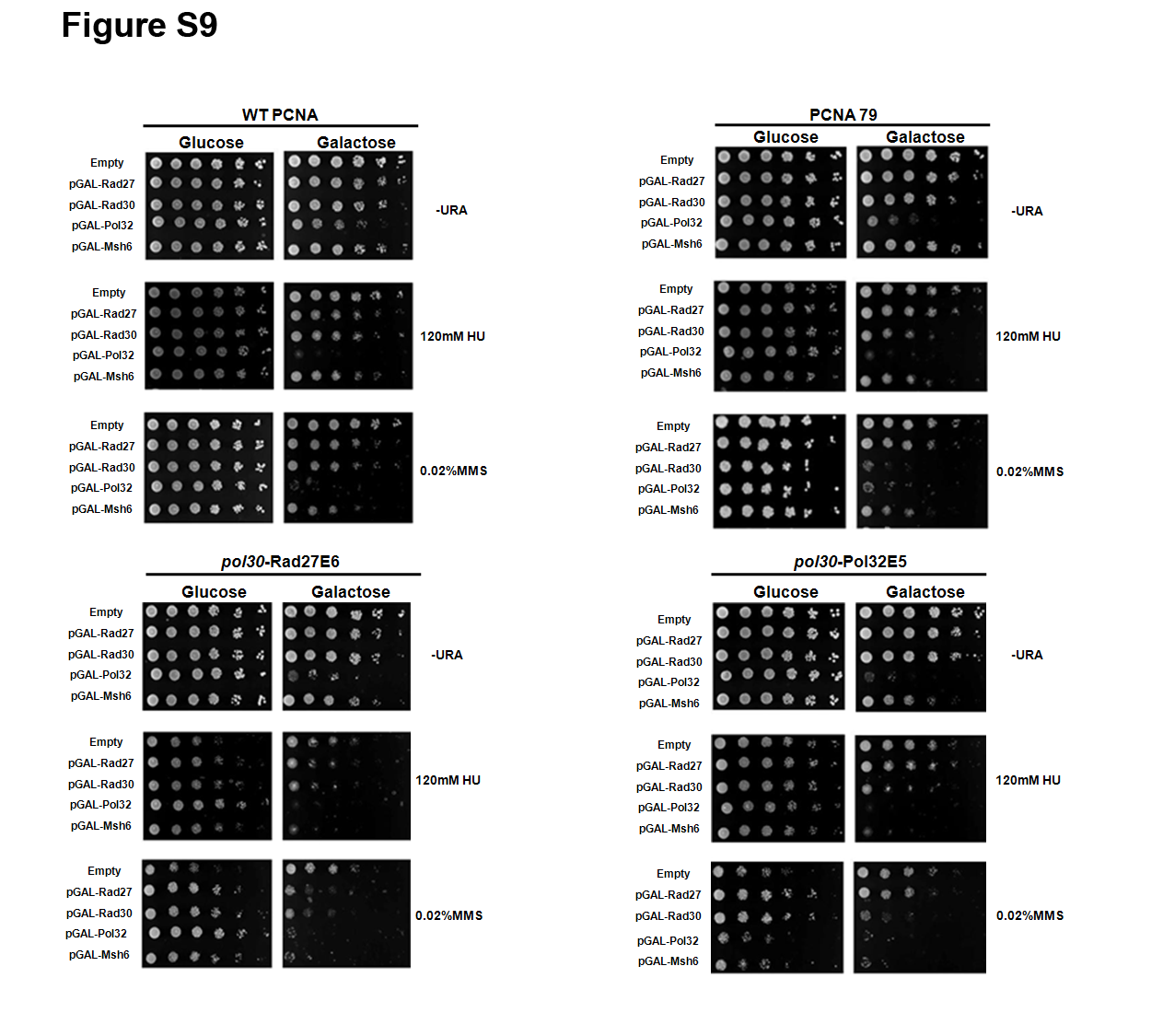

Supplement: Figure S9 — The effects of overexpression of Rad27, Rad30, Pol32, and Msh6 on the growth sensitivity of the POL30 , pol30 -79, pol30 -Rad27E6, or pol30 -Pol32E5 strain. Overexpression of the different partners did not reduce the growth sensitivity of the strains to DNA damaging agents. (0.43 MB TIF) [file pbio.1000507.s009.tif]
