# Supplementary material for: Subtle Alterations in PCNA-Partner Interactions Severely Impair DNA Replication and Repair
Source: PLoS Biol. 2010 Oct 12;8(10):e1000507. doi: 10.1371/journal.pbio.1000507 (PMC2953525; doi:10.1371/journal.pbio.1000507)
Supplement: Table S1 — Yeast two hybrid analysis of binding of selected PCNA mutants with seven additional partners not described in Table 1 of the main text. (0.01 MB DOC) [file pbio.1000507.s010.pdf]

Table S1: Yeast two hybrid analysis of binding of selected PCNA mutants with seven additional partners not described in Table 1 of the main text.

**Mutant**[a,b]	**Partners**[b]						
	**Cdc9**	**Eco1**	**Msh3**	**Rfc1**	**Cac1**	**Rrm3**	**Apn2**
pol30-Rad30E2	WT	WT	WT	WT	WT	WT	-
pol30-Rad30E9	WT	WT	WT	WT	WT	WT	WT
pol30-Rad27E6	WT	WT	WT	WT	+	WT	WT
pol30-Rad27E31	WT	WT	WT	WT	+	WT	WT
pol30-Rad27L1	WT	--	WT	WT	--	WT	WT
pol30-Rad27L2	WT	--	WT	WT	--	WT	WT
pol30-Pol32E5	WT	WT	WT	WT	WT	WT	WT
pol30-Pol32E9	-	WT	WT	WT	WT	WT	WT
pol30-Msh6E2	WT	WT	+	WT	WT	WT	WT
pol30-Msh6E6	WT	WT	++	WT	WT	WT	WT
pol30-Ung1E2	WT	--	WT	WT	ND	ND	WT
pol30-Ung1E3	WT	-	WT	WT	WT	WT	WT
pol30-79	---	---	---	-	---	---	---

[a] PCNA mutants with enhanced affinities for the different target partners (see Table 1, text).

[b] PCNA mutants were fused to the activating domain (AD), while the different partners were fused to the DNA-binding domain (BD). The growth of the resulting Y2H strains was compared to the growth of the WT PCNA-expressing strain following spotting onto selective agar plates (see Material and Methods). Growth rates on the agar plates were scored and tabulated as follows: ++ major increase in growth ; + moderate increase in growth; WT-similar growth as the WT PCNA-expressing cells; - minor decrease in growth; -- moderate decrease in growth; --- major decrease in growth. ND-Not determined.
