# Supplementary material for: Subtle Alterations in PCNA-Partner Interactions Severely Impair DNA Replication and Repair
Source: PLoS Biol. 2010 Oct 12;8(10):e1000507. doi: 10.1371/journal.pbio.1000507 (PMC2953525; doi:10.1371/journal.pbio.1000507)
Supplement: Table S2 — CAN1 mutation spectra for POL30 mutants. (0.08 MB DOC) [file pbio.1000507.s011.doc]

**Table 3**: *CAN1* mutation spectra for *POL30* mutants

| Mutation | Frequency (%) | Type of event | Genotype |
| --- | --- | --- | --- |
| 67bp^a^ | 7/37 (19) | deletions | *pol30*-Pol32E5 |
|  | 9/37 (24) | frameshifts |
| T_6_→T_5_ | 1/37 |
| T_5_→T_4_ | 1/37 |
| T_3_→T_4_ | 1/37 |
| T_2_→T_3_ | 1/37 |
| G_2_→G_3_ | 1/37 |
| G_1_→G_2_ | 1/37 |
| G_1_→G_0_ | 1/37 |
| A_2_→A_3_ | 1/37 |
| C_1_→C_0_ | 1/37 |
|  | 21/37 (57) | substitutions |
| G→A | 9/37 |
| G→T | 5/37 |
| G→C | 2/37 |
| T→G | 2/37 |
| C→G | 2/37 |
| C→A | 1/37 |
| 16^b^ | 1/26 (4) | deletions | *pol30*-Rad27E6 |
|  | 11/26 (42) | frameshifts |
| A_2_→A_1_ | 2/11 |
| A_1_→A_0_ | 1/11 |
| A_5_→A_6_ | 1/11 |
| T_1_→T_0_ | 1/11 |
| T_2_→T_1_ | 1/11 |
| T_3_→T_2_ | 1/11 |
| T_3_→T_4_ | 1/11 |
| G_1_→G_0_ | 1/11 |
| G_1_→G_2_ | 1/11 |
| G_2_→G_3_ | 1/11 |
|  | 14/26 (54) | substitutions |
| C→A | 5/14 |
| C→G | 3/14 |
| G→C | 2/14 |
| T→G | 2/14 |
| G→A | 1/14 |
| A→C | 1/14 |
|  | 0/8 (0) | deletions | *pol30*-Pol32E5  ΔRev3 |
|  | 0/8 (0) | frameshifts |
|  | 8/8 (100) | substitutions |
| G→A | 2/8 |
| C→A | 2/8 |
| A→T | 2/8 |
| T→G | 1/8 |
| T→A | 1/8 |
|  | 0/6 | deletions | *pol30*-Pol32E5  K164R |
| G_1_→G_0_ | 1/6 (16) | frameshifts |
|  | 5/6 (84) | substitutions |
| C→T | 2/6 |
| T→A | 2/6 |
| T→C | 1/6 |
|  | 0/6 | deletions | *pol30*-Rad27E6  ΔRev3 |
|  | 0/6 | frameshifts |
|  | 6/6 (100) | substitutions |
| A→T | 6/6 |
|  | 0/8 (0) | deletions | *pol30*-Rad27E6  K164R |
| T_4_→T_5_ | 1/8 (12.5) | frameshfits |
|  | 7/8 (87.5) | substitutions |
| C→A | 2/8 |
| T→C | 2/8 |
| C→T | 1/8 |
| T→A | 1/8 |
| A→T | 1/8 |

^a^**TTCA*ATG****GTGTTAGCTTTGCTGCCGCCTATATCTCTATTTTCCTGTTCTTAGCTGTT TGGATCTTAT* ***TTCA*ATG**

The 67 bp deletion for this mutant is listed in italics. Repetitive flanking sequences are shown in bold.

^b^ TTACATGGAGACA*TCTACTG GTGGTGACA*AAGTTTTCGAA

The 16 bp deletion for this mutant is listed in italics
