# Supplementary material for: Subtle Alterations in PCNA-Partner Interactions Severely Impair DNA Replication and Repair
Source: PLoS Biol. 2010 Oct 12;8(10):e1000507. doi: 10.1371/journal.pbio.1000507 (PMC2953525; doi:10.1371/journal.pbio.1000507)
Supplement: Table S3 — Sequences of oligonucleotides used in this study. (0.05 MB DOC) [file pbio.1000507.s012.doc]

**Table S3: Sequences of oligonucleotides used in this study**

| **Sequence** | **Primer** |
| --- | --- |
| 5'AAAGTGTAAGCGGCCGCGCATTTTTCTTCTCCATCCGACG3' | fr-pro |
| 5'GAAACGCGTACTAGTTTTTTCTCTCTTTTGACTGCGTACTTAG3' | rev-pro |
| 5'GTTAATTTCAAGCTTTAAATGTAAATTATCTATATAGTTGTATACTAAAAATAATAAAC3' | fr-ter |
| 5'AATAATTGACTCGAGGGATGTGGAAACCCTGAATACCAC3' | rev-ter |
| 5'TTTCACTCACAGCAACAAGCAGCAAGCACTAAGTACGCAGTCAAAAGAGAGAAAAAATGTT AGAAGCAAAATTTGAAGAAGCATC3' | fr-pRS/PCNA |
| 5'GTTTTTTTTTTGTTTATTATTTTTAGTATACAACTATATAGATAATTTACATTTTTATTC TTCG TCATTAAATTTAGGAGCC3' | rev-pRS/PCNA |
| 5'CCAAACCCAAAAAAAGAGATCGAATTAATGTTAGAAGCAAAATTTGAAGAAGCATC3' | fr-pAD/PCNA |
| 5'CACTATAGGGCTCTAGAGTCGACTAATACTCTCGAGTTATTCTTCGTCATTAAATTTAGGAGCC3' | rev-pAD/PCNA |
| 5'GGTCAAAGACAGTTGACTGTATCGCCGGAATTCGCCCGGGCCTCGAGCCCGATGTCAAAATTTACTTGGAAGGAGTTG3' | fr-pBD/RAD30 |
| 5'TAATAAAAATCATAAATCATAAGAAATTCGCCCGGAATTAGCTTGGCTGCAGTTTCATTTTTTTCTTGTAAAAAATGATAAG3' | rev-pBD/RAD30 |
| 5'GGTCAAAGACAGTTGACTGTATCGCCGGAATTCGCCCGGGCCTCGAGCCCGATGGGTATTAAAGGTTTGAATGCAATTATATC3' | fr-pBD/RAD27 |
| 5'TAATAAAAATCATAAATCATAAGAAATTCGCCCGGAATTAGCTTGGCTGCAGTTATCATCTTCTTCCCTTTGTGACTTTATTC3' | rev-pBD/RAD27 |
| 5'GGTCAAAGACAGTTGACTGTATCGCCGGAATTCGCCCGGGCCTCGAGCCCGATGGATCAAAAGGCGTCATATTTTATC3' | fr-pBD/POL32 |
| 5'TAATAAAAATCATAAATCATAAGAAATTCGCCCGGAATTAGCTTGGCTGCAGTTTTATTTTGCCTTTCTTTTGAAAAAGC3' | rev-pBD/POL32 |
| 5'GGTCAAAGACAGTTGACTGTATCGCCGGAATTCGCCCGGGCCTCGAGCCCGATGGCCCCAGC TACCCCTAAAAC3' | fr-pBD/MSH6 |
| 5'AAATCATAAATCATAAGAAATTCGCCCGGAATTAGCTTGGCTGCAGTTATTAGGATTGTAAAT CATCAATTATACTAAATAGAC3' | rev-pBD/MSH6 |
| 5'GGTCAAAGACAGTTGACTGTATCGCCGGAATTCGCCCGGGCCTCGAGCCCGATGTGGTGCATGAG AAGATTGCC3' | fr-pBD/UNG1 |
| 5'TAATAAAAATCATAAATCATAAGAAATTCGCCCGGAATTAGCTTGGCTGCAGTTATCAAGGG TCCTTTGATTCTGACTC3' | rev-pBD/UNG1 |
| 5'GGTCAAAGACAGTTGACTGTATCGCCGGAATTCGCCCGGGCCTCGAGCCCGATGGCGGGACA ACCCACAATAAG3' | fr-pBD/MSH3 |
| 5'TAATAAAAATCATAAATCATAAGAAATTCGCCCGGAATTAGCTTGGCTGCAGTTATCAGTGG ATATCCAATGATAGTAATTTCG3' | rev-pBD/MSH3 |
| 5'GGTCAAAGACAGTTGACTGTATCGCCGGAATTCGCCCGGGCCTCGAGCCCGATGGTCAATA TTTCTGATTTCTTTGG3' | fr-pBD/RFC1 |
| 5'TAATAAAAATCATAAATCATAAGAAATTCGCCCGGAATTAGCTTGGCTGCAGTTATCATGCTT TCGTTTTCCTTTTTTTG3' | rev-pBD/RFC1 |
| 5'GGTCAAAGACAGTTGACTGTATCGCCGGAATTCGCCCGGGCCTCGAGCCCGATGAAAGCTAGGAAATCGCAGAGAAAAG3' | fr-pBD/ECO1 |
| 5'TAATAAAAATCATAAATCATAAGAAATTCGCCCGGAATTAGCTTGGCTGCAGTTATCATATGTATACCGGCAATAGTAACTTG3' | rev-pBD/ECO1 |
| 5'GGTCAAAGACAGTTGACTGTATCGCCGGAATTCGCCCGGGCCTCGAGCCCGATGGAGCAACATCTCAAATCAATTC3' | fr-pBD/CAC1 |
| 5'TAATAAAAATCATAAATCATAAGAAATTCGCCCGGAATTAGCTTGGCTGCAGTTATTACAAAGACGGGGTTGGCATATTTG3' | rev-pBD/CAC1 |
| 5'GGTCAAAGACAGTTGACTGTATCGCCGGAATTCGCCCGGGCCTCGAGCCCGATGCGCAGATTACTGACCGGTTG3' | fr-pBD/CDC9 |
| 5'TAATAAAAATCATAAATCATAAGAAATTCGCCCGGAATTAGCTTGGCTGCAGTTACTAATTTTGCATGTGGGATTGGTTTTC3' | rev-pBD/CDC9 |
| 5'GGTCAAAGACAGTTGACTGTATCGCCGGAATTCGCCCGGGCCTCGAGCCCGATGTTCAGGTCGCATGCCTCC3' | fr-pBD/RRM3 |
| 5'TAATAAAAATCATAAATCATAAGAAATTCGCCCGGAATTAGCTTGGCTGCAGTCATTTCAAAGTTTCTAAACGTTTATAGAAATC3' | rev-pBD/RRM3 |
| 5'GGTCAAAGACAGTTGACTGTATCGCCGGAATTCGCCCGGGCCTCGAGCCCGATGTCATCAAGCGAAAACACGTTAC3' | fr-pBD/APN2 |
| 5'TAATAAAAATCATAAATCATAAGAAATTCGCCCGGAATTAGCTTGGCTGCAGTTATTAAACCCACTGAAAAAACCCACAAG3' | rev-pBD/RRM3 |
| 5'CGACGATTGAAGGTAGATACCCATACGACGTTCCAGACTACGCTCTGCAG | fr-Lib1 |
| 5'CCATCAATTTCAGAGAGTATTCGGC3' | rev-NNS |
| 5'GCCGAATACTCTCTGAAATTGATGGATNNSGATNNSGATNNSTTAAAGATTNNSGAATTACA GTACGACTCCACCCTGTCATTG3' | fr-NNS |
| 5'CAGATCTCGAGCTATTACAAGTCCTCTTCAGAAATAAGCTTTTGTTC3' | rev-Lib1 |
| 5'ATTCGATGCATATGTTAGAAGCAAAATTTGAAGAAGC3' | fr-pET/PCNA |
| 5'TCACTCAGCTCGAGCTATTATTCTTCGTCATTAAATTTAGGAGCC3' | rev-pET/PCNA |
| 5'GACAGCATACATTGGAAAGAAATAGGAAACGGACACCGGAAGAAAAAATAGATCTGTTTAG CTTGCCTTGTCCC3' | fr-Rad27::hyg |
| 5'ATATATGCCAAGGTGAAGGACCAAAAGAAGAAAGTGGAAAAAGAACCCCCGAGCTCGTTTTC GACACTGGATGG3' | rev-Rad27::hyg |
